# Loss of soluble guanylyl cyclase in platelets contributes to atherosclerotic plaque formation and vascular inflammation

**DOI:** 10.1038/s44161-022-00175-w

**Published:** 2022-12-12

**Authors:** Carina Mauersberger, Hendrik B. Sager, Jana Wobst, Tan An Dang, Laura Lambrecht, Simon Koplev, Marlène Stroth, Noomen Bettaga, Jens Schlossmann, Frank Wunder, Andreas Friebe, Johan L. M. Björkegren, Lisa Dietz, Sanne L. Maas, Emiel P. C. van der Vorst, Peter Sandner, Oliver Soehnlein, Heribert Schunkert, Thorsten Kessler

**Affiliations:** 1 German Heart Centre Munich, Department of Cardiology, Technical University of Munich, Munich, Germany.; 2 German Centre for Cardiovascular Research, Munich Heart Alliance, Munich, Germany.; 3 Department of Genetics and Genomic Sciences, Icahn Institute for Genomics and Multiscale Biology, Icahn School of Medicine at Mount Sinai, New York, NY, USA.; 4 Cancer Research UK Cambridge Institute, University of Cambridge, Li Ka Shing Centre, Cambridge, UK.; 5 Department of Pharmacology and Toxicology, University of Regensburg, Regensburg, Germany.; 6 Bayer AG, R&D Pharmaceuticals, Wuppertal, Germany.; 7 Institute of Physiology, Julius Maximilian University of Würzburg, Würzburg, Germany.; 8 Department of Medicine, Neo, Karolinska Institutet, Karolinska Universitetssjukhuset, Huddinge, Sweden.; 9 Department of Cardiac Surgery and The Heart Clinic, Tartu University Hospital and Department of Cardiology, Institute of Clinical Medicine, Tartu University, Tartu, Estonia.; 10 Institute for Molecular Cardiovascular Research and Interdisciplinary Centre for Clinical Research, Rhine-Westphalia Technical University of Aachen, Aachen, Germany.; 11 Institute for Cardiovascular Prevention, Ludwig Maximilian University of Munich, Munich, Germany.; 12 Institute for Experimental Pathology, University of Münster, Münster, Germany.; 13 Department of Physiology and Pharmacology and Department of Medicine, Karolinska Institutet, Stockholm, Sweden.; 14 These authors contributed equally: Carina Mauersberger, Hendrik B. Sager.; 15 These authors jointly supervised this work: Heribert Schunkert, Thorsten Kessler.

## Abstract

Variants in genes encoding the soluble guanylyl cyclase (sGC) in platelets are associated with coronary artery disease (CAD) risk. Here, by using histology, flow cytometry and intravital microscopy, we show that functional loss of sGC in platelets of atherosclerosis-prone *Ldlr*^−/−^ mice contributes to atherosclerotic plaque formation, particularly via increasing in vivo leukocyte adhesion to atherosclerotic lesions. In vitro experiments revealed that supernatant from activated platelets lacking sGC promotes leukocyte adhesion to endothelial cells (ECs) by activating ECs. Profiling of platelet-released cytokines indicated that reduced platelet angiopoietin-1 release by sGC-depleted platelets, which was validated in isolated human platelets from carriers of *GUCY1A1* risk alleles, enhances leukocyte adhesion to ECs. I mp or ta ntly, p ha rm ac ol ogical sGC stimulation increased platelet angiopoietin-1 release in vitro and reduced leukocyte recruitment and atherosclerotic plaque formation in atherosclerosis-prone *Ldlr*^−/−^ mice. Therefore, pharmacological sGC stimulation might represent a potential therapeutic strategy to prevent and treat CAD.

CAD is a leading cause of morbidity and mortality in industrialized nations^1^. In the past 15 years, large-scale genetic studies led to the identification of more than 300 genomic variants that are associated with CAD and myocardial infarction (MI) risk. Interestingly, most of the affected genes do not influence traditional risk factors^2^ but rather mechanisms not previously implicated in the pathophysiology of atherosclerosis. One prominent example is the nitric oxide (NO)–cyclic guanosine monophosphate (cGMP) signaling pathway, which contains several genome-wide significantly associated variants for CAD^3^. In the center of this pathway sits sGC, which is activated by NO and produces the second messenger cGMP. From a genetic perspective, private mutations in the genes encoding for sGC and rare coding and common noncoding variants in the *GUCY1A1* gene, which encodes the α_1_-subunit of the sGC, were all associated with CAD or premature MI by exome sequencing and genome-wide association studies (GWAS), respectively^4,5^. These mutations and variants reduce sGC expression or activity^5–7^; in line with this, enhanced NO–cGMP signaling has been associated with reduced risk of several cardiometabolic phenotypes including CAD and peripheral artery disease^8^.

*GUCY1A1* and *GUCY1B1*, genes encoding sGC, are expressed at high levels in platelets and vascular smooth muscle cells. In humans, carriers of the CAD risk variant rs7692387 have reduced sGC α_1_ protein levels, which might impair the effects of the natural platelet inhibitor NO^7^. Indeed, a retrospective analysis of two randomized trials revealed that inhibition of platelet activity by aspirin successfully reduced cardiovascular events in primary prevention only in homozygous carriers of the *GUCY1A1* risk allele^9^. Since the overall role of platelets in atherosclerosis is controversial^10^, we decided to delete sGC in mice platelets specifically by knockout of its β_1_-subunit to investigate the contribution of platelet sGC on atherosclerosis and vascular inflammation and further evaluate the potential of sGC stimulation as a therapeutic strategy.

## Results

### Atherosclerotic plaque formation in mice lacking platelet sGC

**Extended Data Fig. 1 |:**
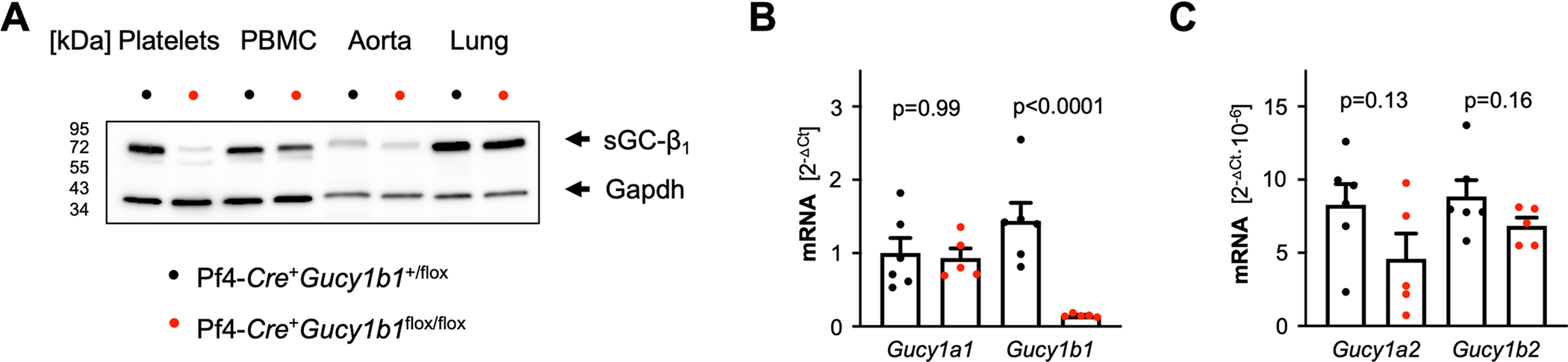
sGC-Expression in Pf4-*Cre*^+^*Gucy1b1*^*LoxP/LoxP*^ compared to Pf4-*Cre*^+^*Gucy1b1*^+/LoxP^ mice. **a**. Expression of sGC-β_1_ in platelets, peripheral blood mononuclear cells (PBMC), aorta, and lung. Representative of three independently performed Western blots on different samples. **B**+**C**. *Gucy* transcript expression analysis in megakaryocytes of Pf4-*Cre*^+^*Gucy1b1*^+/*LoxP*^ and Pf4-*Cre*^+^*Gucy1b1*^*LoxP/LoxP*^ mice. **b**. *Gucy1a1* and *Gucy1b1*. **C**. *Gucy1a2* and *Gucy1b2*. Each symbol represents one independent animal (n = 6 for control group, n = 5 for Pf4-*Cre*^+^*Gucy1b1*^*LoxP/LoxP*^). Two-sided unpaired t-test. Data are mean and s.e.m.

**Extended Data Fig. 2 |:**
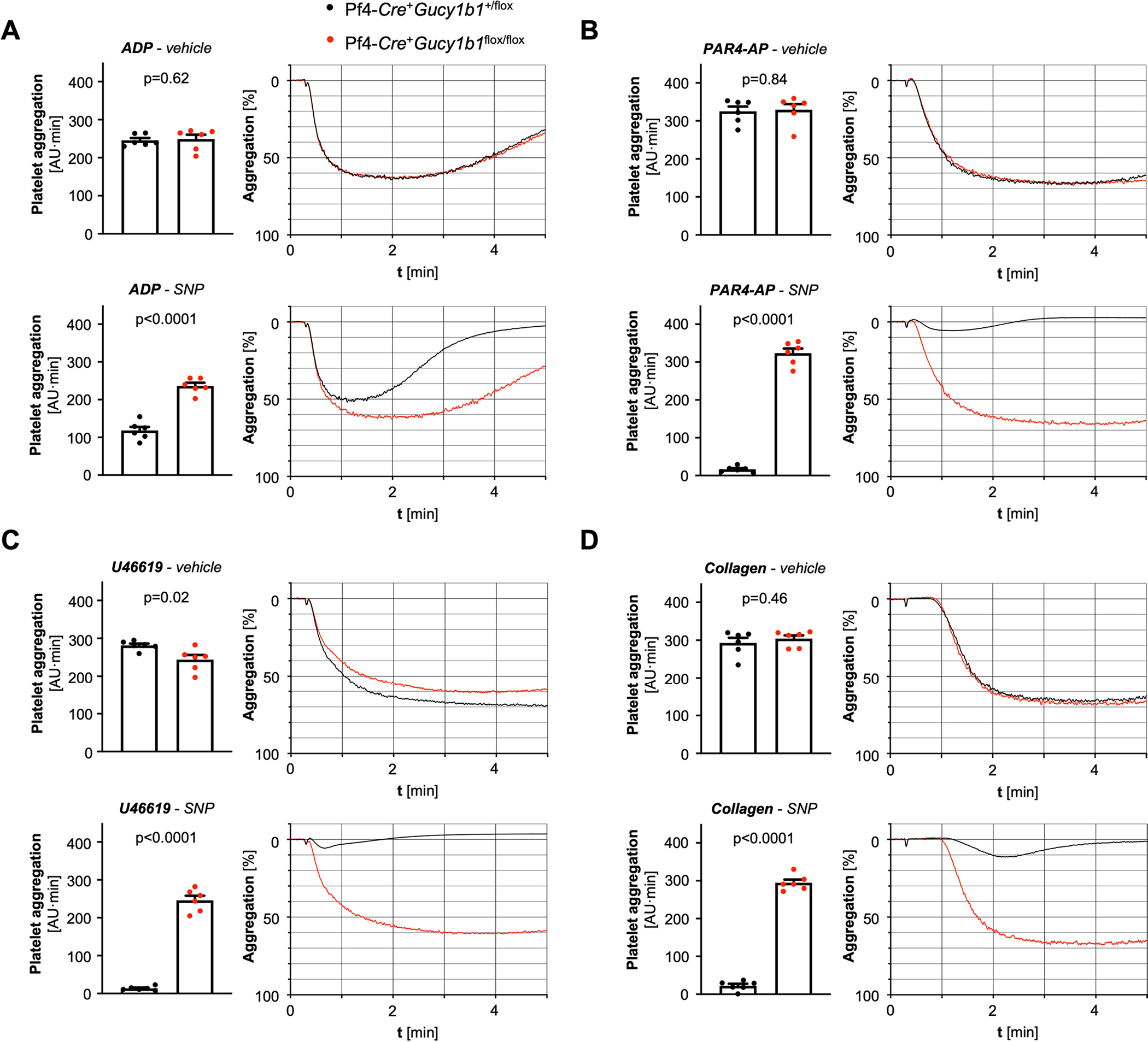
Platelet aggregation in control and platelet sGC knockout (Pf4-*Cre*^+^*Gucy1b1*^*LoxP/LoxP*^) mice. Platelet aggregation in control and platelet sGC knockout (Pf4-*Cre*^+^*Gucy1b1*^*LoxP/LoxP*^) mice after stimulation with adenosine diphosphate (ADP, **a**) the platelet-activated receptor 4 agonist PAR4-AP (**b**), the thromboxane analog U46619 (**c**), and collagen (**d**). Each experiment was performed in the presence of DMSO (vehicle) or the nitric oxide donor sodium nitroprusside (SNP). Each symbol represents one independent animal (n = 6). Aggregation tracings represent the mean values of investigated animals per genotype. Two-sided unpaired t-test (except **A**, vehicle and **D**, vehicle: two-sided Mann-Whitney U-test). Data are mean and s.e.m.

**Extended Data Fig. 3 |:**
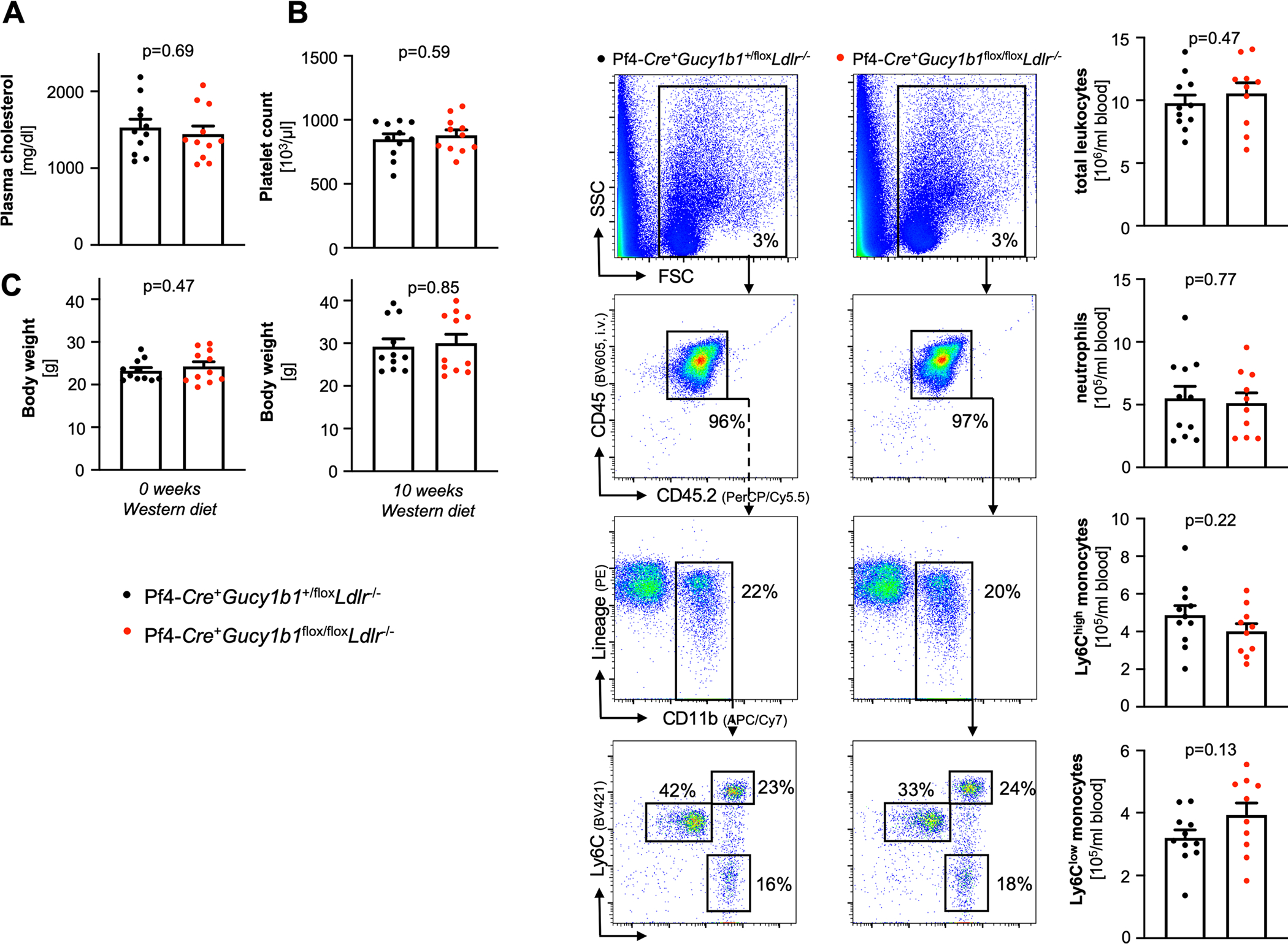
Pf4-*Cre*^+^*Gucy1b1*^*LoxP/LoxP*^*Ldlr*^−/−^ compared to Pf4-*Cre*^+^*Gucy1b1*^+/LoxP^*Ldlr*^−/−^ mice after Western diet (see Fig. 1e). **a**. Serum cholesterol levels (n = 11). **b**. Platelet count (n = 11). **c**. Body weight (n = 11) at baseline (left) and after ten weeks (right). **d**. Blood leukocyte numbers and subsets (n = 11 for controls, n = 10 for Pf4-*Cre*^+^*Gucy1b1*^*LoxP/LoxP*^*Ldlr*^−/−^). Each symbol represents one independent animal. Two-sided unpaired t-test (except **C** 10 weeks: two-sided Mann-Whitney U-test). One outlier was removed in the analysis of **D** according to the ROUT method. Data are mean and s.e.m.

**Extended Data Fig. 4 |:**
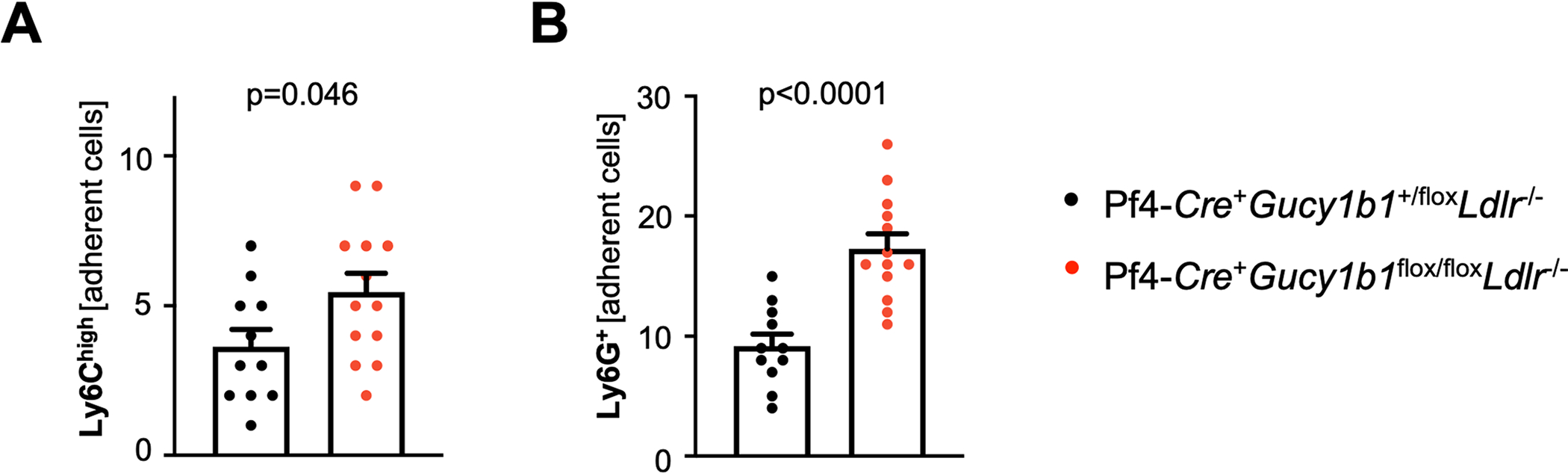
Leukocyte adhesion to atherosclerotic plaques of Pf4-*Cre*^+^*Gucy1b1*^*LoxP/LoxP*^*Ldlr*^−/−^ (n = 13) compared to Pf4-*Cre*^+^*Gucy1b1*^+/LoxP^*Ldlr*^−/−^ mice (n = 11) after Western diet assessed by fluorescence intravital microscopy. **a**. Ly6C^high^ monocytes. **b**. Neutrophils. Each symbol represents one independent animal. Two-sided unpaired t-test. Data are mean and s.e.m.

**Fig. 1 |:**
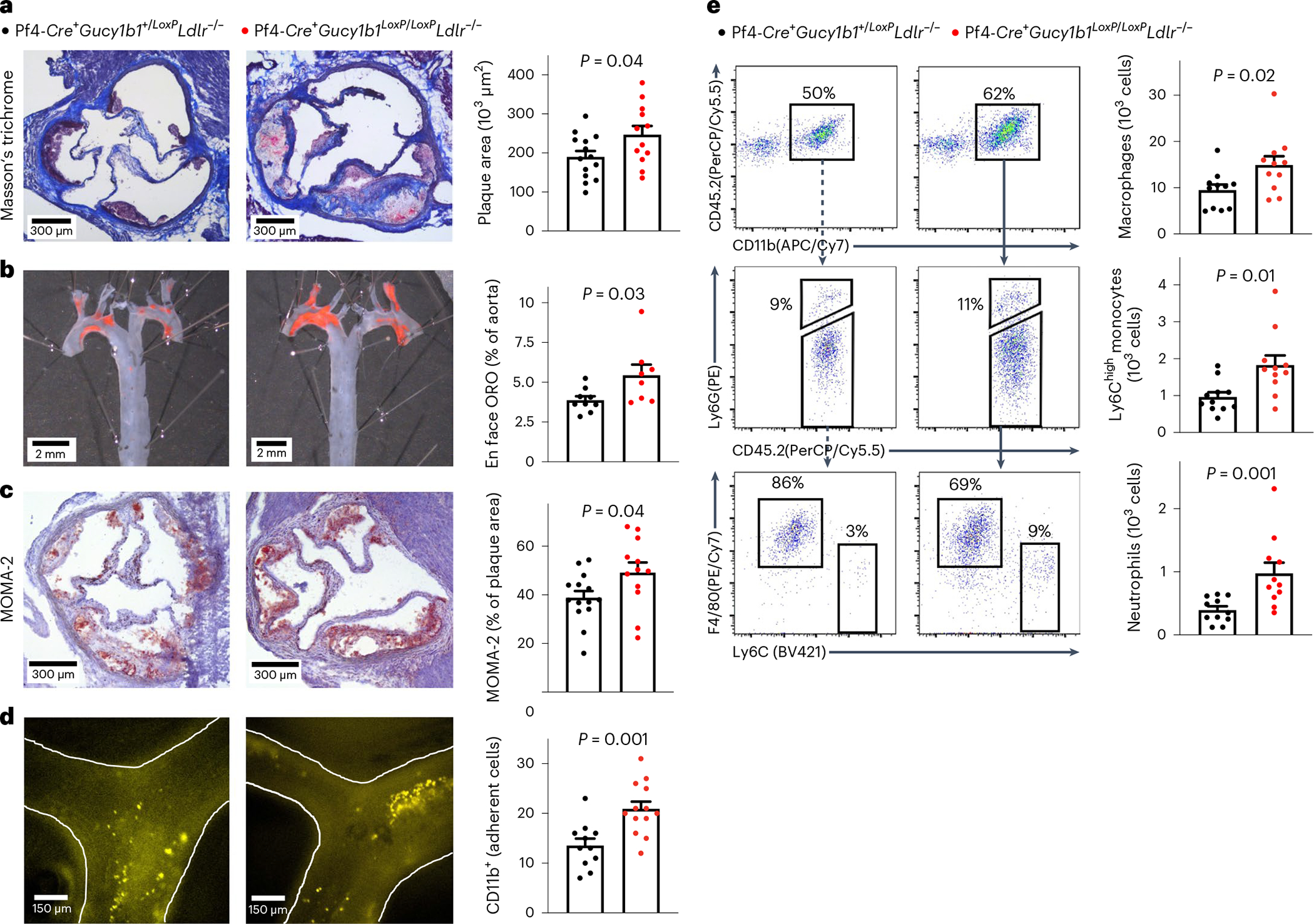
Atherosclerotic plaque formation and vascular inflammation in mice lacking platelet sGC. **a**–**c**, Atherosclerotic plaque formation as assessed by aortic root histology (**a**), aortic en face ORO staining (**b**), and monocyte and macrophage content (**c**) in 12 (**b**: 8) Pf4-*Cre*^+^*Gucy1b1*^*LoxP*/*LoxP*^*Ldlr*^−/−^ mice compared to 14 (**b**: 9) Pf4-*Cre*^+^*Gucy1b1*^+/*LoxP*^*Ldlr*^−/−^ mice that were fed a Western diet for 10 weeks. Two-sided unpaired *t*-test. **d**, Leukocyte adhesion to atherosclerotic plaques in *n* = 13 Pf4-*Cre*^+^*Gucy1b1*^*LoxP*/*LoxP*^*Ldlr*^−/−^ mice compared to *n* = 11 Pf4-*Cr e*^+^*Gucy1b1*^+/*LoxP*^*Ldlr*^−/−^ mice that were fed a Western diet for 6 weeks to induce atherosclerotic plaque formation. Two-sided unpaired *t*-test. **e**, Quantification of vascular inflammation by flow cytometry analysis of aortic cell suspensions of Pf4-*Cre*^+^*Gucy1b1*^*LoxP*/*LoxP*^*Ldlr*^−/−^ mice compared to Pf4-*Cre*^+^*Gucy1b1*^+/*LoxP*^*Ldlr*^−/−^ mice (*n* = 11 per group). Two-sided Mann–Whitney *U*-test. Each symbol represents one independent animal. Data are the mean ± s.e.m.

We created mice with a platelet-predominant sGC knockout (Pf4-*Cre*^+^*Gucy1b1*^*LoxP*/*LoxP*^) which displayed reduced sGC β_1_ protein levels compared to wild-type (WT) platelet sGC mice (Extended Data Fig. 1a); expectedly, this reduced inhibition of agonist-induced platelet aggregation by the NO donor sodium nitroprusside (Extended Data Fig. 2). Importantly, the expression of other sGC subunits was not altered (Extended Data Fig. 1b,c). To examine the effect of platelet sGC on atherosclerosis, we crossbred these animals with atherosclerosisprone mice (*Ldlr*^−/−^ mice) which were then fed a Western diet for ten weeks. Body weight, serum cholesterol, and hematological parameters were comparable between the genotypes (Extended Data Fig. 3). Pf4-*Cre*^+^*Gucy1b1*^*LoxP*/*LoxP*^*Ldlr*^−/−^ mice displayed larger plaques in the aortic root (246,998 ± 22,162 μm^2^ (*n* = 12) versus 189,843 ± 15,156 μm^2^ (*n* = 14), *P* = 0.04; Fig. 1a) and aorta en face analysis (5.4 ± 0.7% (*n* = 8) versus 3.9 ± 0.2% (*n* = 9), *P* = 0.03; Fig. 1b). Immunohistochemical staining of aortic root sections revealed increased plaque monocyte and macrophage content in Pf4-*Cre*^+^*Gucy1b1*^*LoxP*/*LoxP*^*Ldlr*^−/−^ mice compared to Pf4-*Cre*^+^*Gucy1b1*^+/*LoxP*^*Ldlr*^−/−^ mice (49.1 ± 4.2% (*n* = 12) versus 38.8 ± 2.8% (*n* = 14) of plaque area, *P* = 0.04; Fig. 1c). To explore why monocyte/macrophages accumulated more in mice with platelet-predominant sGC depletion, we performed intravital fluorescence microscopy of carotid artery plaques in Pf4-*Cre*^+^*Gucy1b1*^*LoxP*/*LoxP*^*Ldlr*^−/−^ and Pf4-Cre^+^*G ucy1b1*^+/*LoxP*^*Ldlr*^−/−^ mice which were fed a Western diet for six weeks to initiate plaque formation. We found enhanced adhesion of leukocytes in Pf4-*Cre*^+^*Gucy1b1*^*LoxP*/*LoxP*^*Ldlr*^−/−^ compared to Pf4-*Cre*^+^*Gucy1b1*^+/*LoxP*^*Ldlr*^−/−^ mice (20.9 ± 1.5 (*n* = 13) versus 13.6 ± 1.4 (*n* = 11) Cd11b^+^ cells, *P* = 0.001; Fig. 1d and Extended Data Fig. 4). Flow cytometry of aortic cell suspensions revealed more numerous Ly6C^high^ monocytes (1,829 ± 262 versus 964 ± 129 cells per aorta, *n* = 11 each, *P* = 0.01), neutrophils (974 ± 170 versus 392 ± 60 cells per aorta, *n* = 11 each, *P* = 0.001) and macrophages (14,895 ± 1,912 versus 9,463 ± 1,211 cells per aorta, *n* = 11 each, *P* = 0.02; Fig. 1e) in Pf4-*Cre*^+^*Gucy1b1*^*LoxP*/*LoxP*^*Ldlr*^−/−^ compared to Pf4-*Cre*^+^*Gucy1b1*^+/*LoxP*^*Ldlr*^−/−^ mice while blood leukocyte numbers were unchanged (Extended Data Fig. 3d). Taken together, these data indicate that the lack of sGC in platelets contributes to vascular inflammation and hence atherosclerotic plaque progression.

### Platelet sGC and leukocyte adhesion in vitro

**Extended Data Fig. 5 |:**
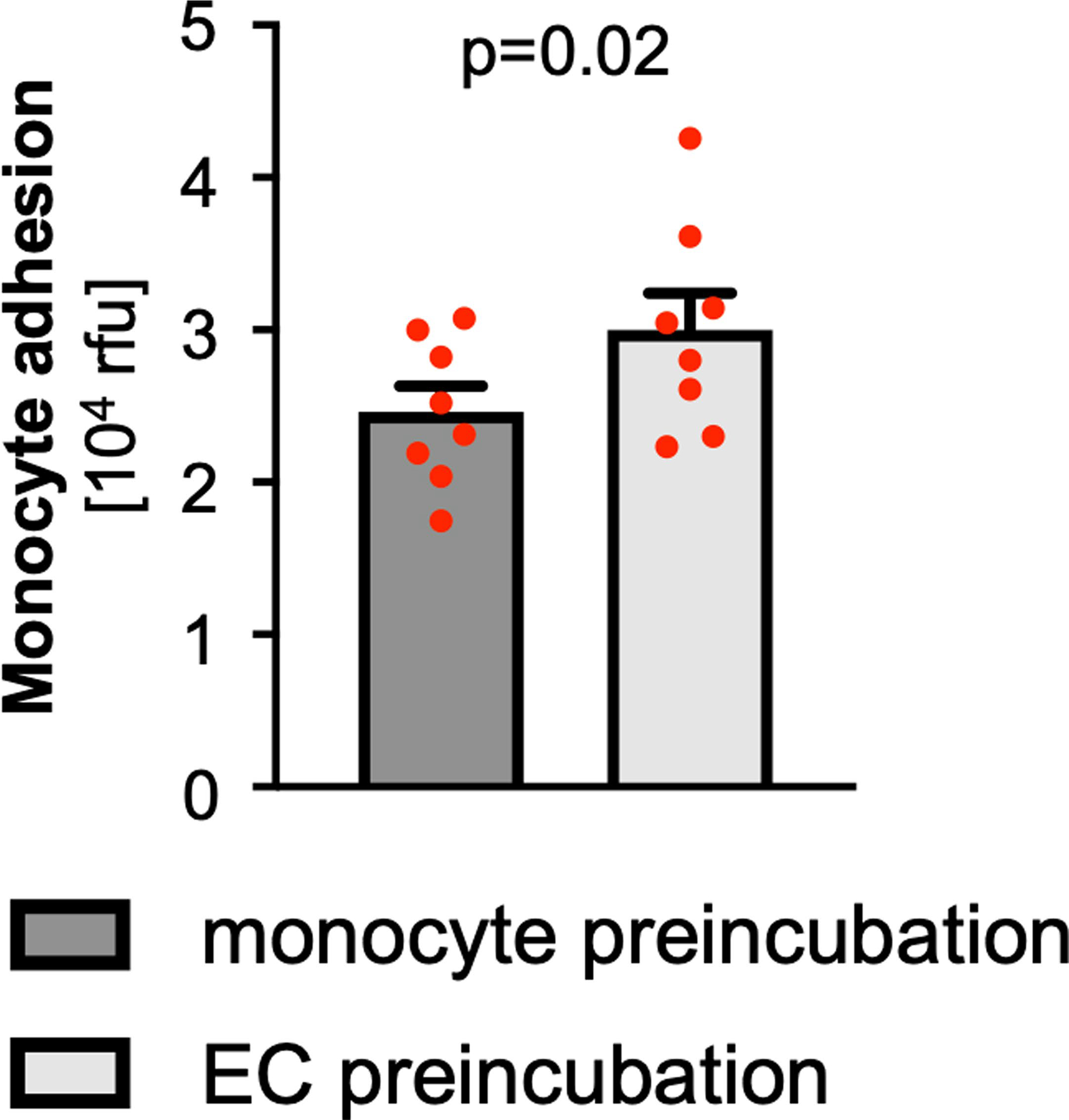
Quantification of monocyte adhesion after preincubation of either EC or monocytes with supernatant of activated platelets from Pf4-*Cre* + *Gucy1b1*^*LoxP/LoxP*^ mice. Each symbol represents one paired sample (derived from n = 8 independent animals). Two-sided paired t-test. Data are mean and s.e.m. Abbreviations: *EC*, endothelial cells; *rfu*, relative fluorescence units.

**Extended Data Fig. 6 |:**
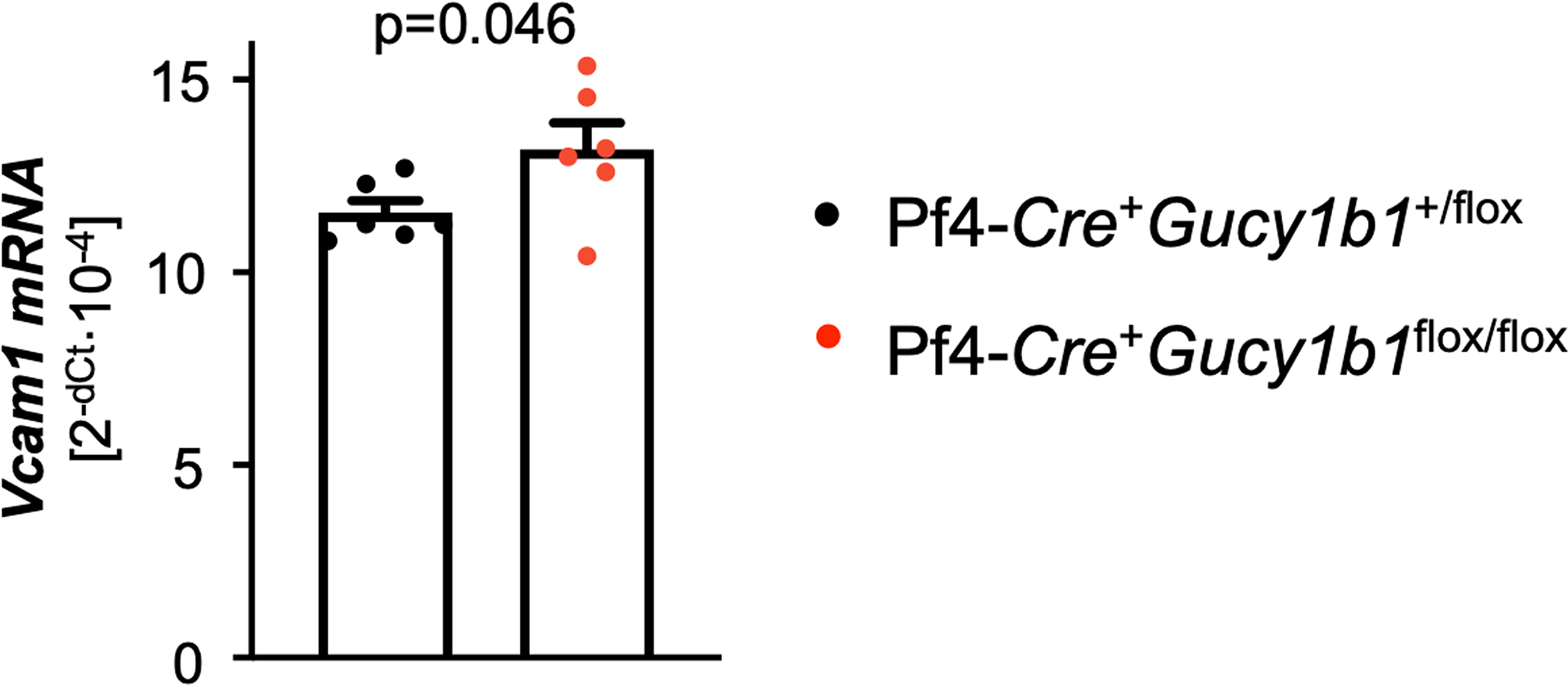
*Vcam1* expression of wild-type endothelial cells after incubation with supernatant of activated Pf4-*Cre*^+^*Gucy1b1*^+/LoxP^ or Pf4-*Cre*^+^*Gucy1b1*^*LoxP/LoxP*^ platelets. Each symbol represents one independent animal (n = 6). Data are mean and s.e.m. Two-sided paired t-test.

**Fig. 2 |:**
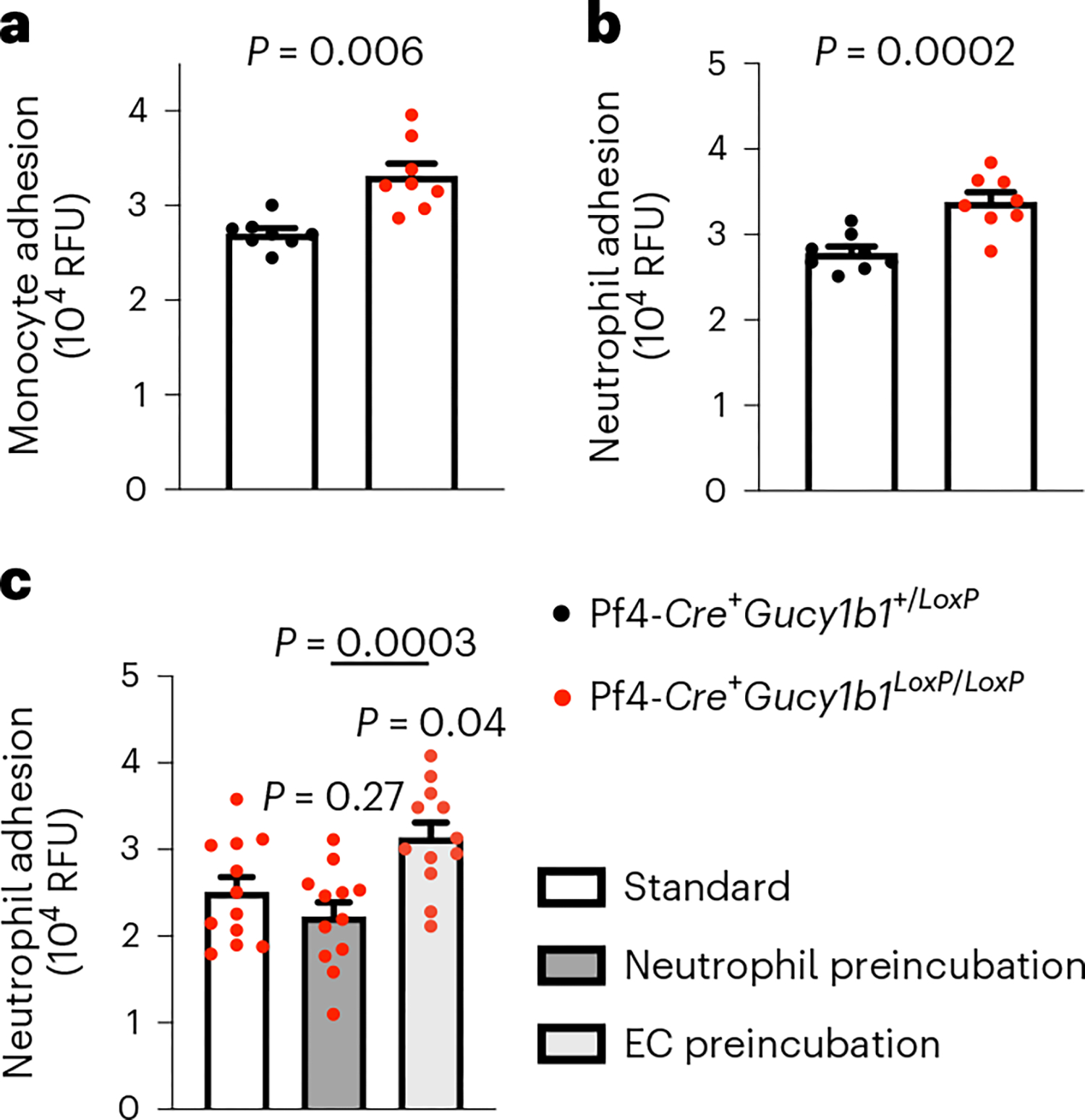
Influence of platelet sGC on leukocyte adhesion in vitro. **a**,**b**, WT monocyte (**a**) and neutrophil (**b**) adhesion to WT ECs after incubation with supernatant of activated platelets isolated from either Pf4-*Cre*^+^*Gucy1b1*^*LoxP*/*LoxP*^ or Pf4-*Cre*^+^*Gucy1b1*^+/*LoxP*^ mice. Each symbol represents 1 independent animal (*n* = 8 per group). Two-sided unpaired *t*-test. Data are the mean ± s.e.m. **c**, Quantification of neutrophil adhesion after preincubation of either ECs or neutrophils with supernatant of activated platelets from Pf4-*Cre*^+^*Gucy1b1*^*LoxP*/*LoxP*^ mice in comparison to non-preincubation conditions. Each symbol represents 1 paired sample (each derived from *n* = 8 independent animals). Repeated measures one-way ANOVA with Tukey test for multiple testing. Data are the mean ± s.e.m.

To follow up on enhanced adhesion of leukocytes to atherosclerotic plaques in mice lacking platelet sGC under proatherogenic conditions, we tested whether this phenotype can be resembled in vitro. Therefore, we isolated blood monocytes and neutrophils from WT mice and incubated these with WT aortic ECs in the presence of activated platelet releasate from Pf4-*Cre*^+^*Gucy1b1*^+/*LoxP*^ or Pf4-*Cre*^+^*Gucy1b1*^*LoxP*/*LoxP*^ mice. We found that incubation with the supernatant of activated platelets lacking sGC enhanced leukocyte, particularly monocyte (33,125 ± 1,313 versus 27,039 ± 555 relative fluorescence units (RFU), *P* = 0.006, *n* = 8; Fig. 2a) and neutrophil (33,810 ± 1,139 versus 27,824 ± 758 RFU, *P* < 0.001, *n* = 8; Fig. 2b) adhesion. To delineate whether leukocytes or ECs are activated by the sGC knockout platelet releasate, we preincubated neutrophils or monocytes and ECs with activated platelet supernatant from Pf4-*Cre*^+^*G ucy1b1*^*LoxP*/*LoxP*^ mice before performing the adhesion assay. We found that preincubation of ECs with supernatant from activated sGC knockout platelets increased adhesion compared to preincubation of neutrophils (31,383 ± 1,731 versus 22,254 ± 1,662 RFU, *P*_adj_ < 0.001, *n* = 12 experiments; Fig. 2c) or monocytes (Extended Data Fig. 5). In line with this, already at this very early time point, we found enhanced expression of the adhesion molecule *Vcam1* in ECs that were incubated with supernatant of activated Pf4-*Cre*^+^*Gucy1b1*^*LoxP*/*LoxP*^ platelets (Extended Data Fig. 6). These data indicate (1) that platelets from Pf4-*Cre*^+^*Gucy1b1*^+/*LoxP*^ and Pf4-*Cre*^+^*Gucy1b1*^*LoxP*/*LoxP*^ mice differentially release a soluble factor and (2) that this preferentially leads to activation of ECs.

### Reduced release of angiopoietin-1 by sGC-deficient platelets

**Extended Data Fig. 7 |:**
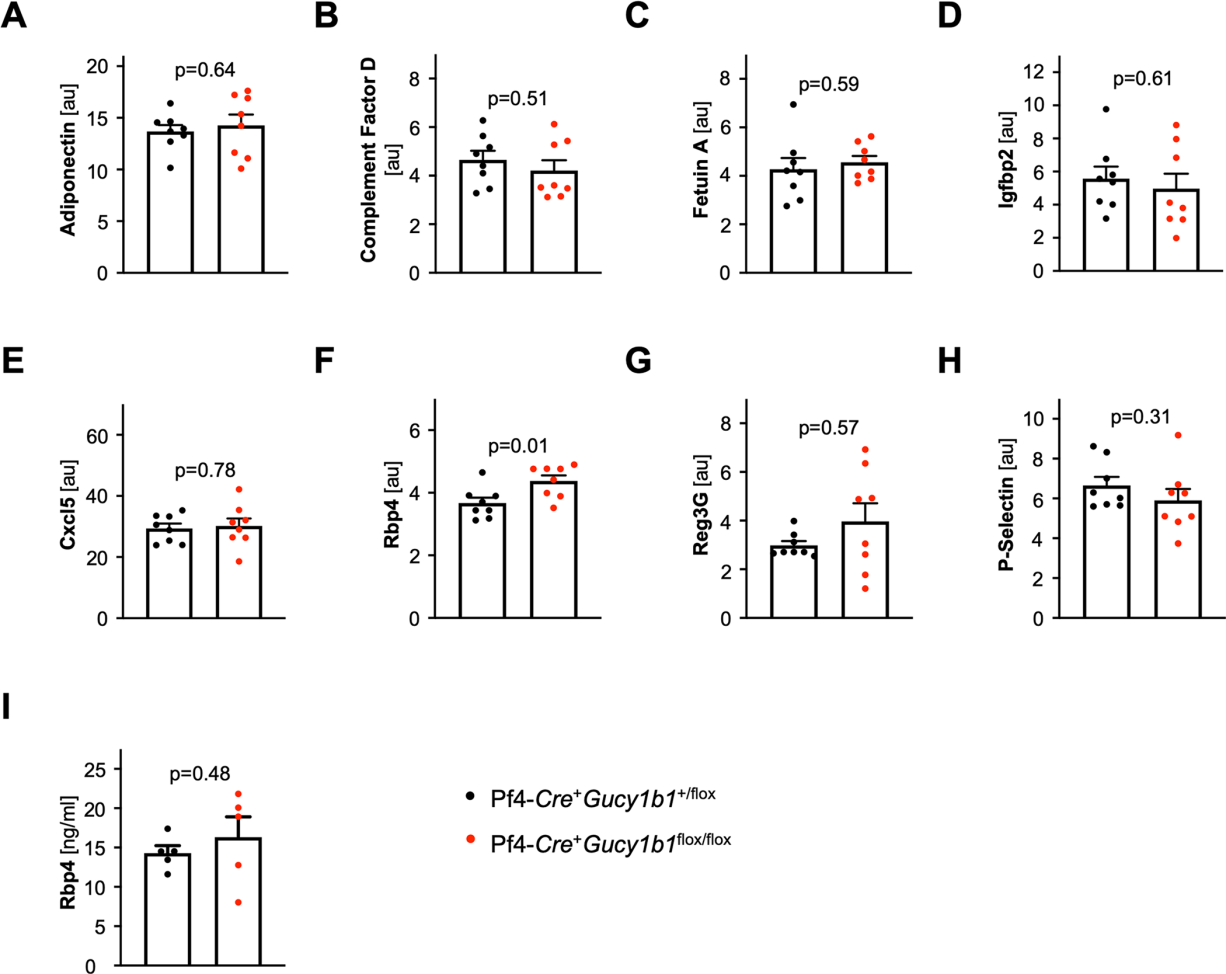
Cytokine profiling. **a**-**h**. Cytokine profiling assay results for cytokines detected at a mean relative intensity ≥1 (in addition to Angiopoietin-1). **I**. Replication attempt for RBP4 release using ELISA. Each symbol represents one independent animal (**A**-**H**: n = 8; **I**: n = 5). Two-sided unpaired t-test (except **B** and **G**: Two-sided Mann-Whitney U-test). Data are mean and s.e.m. Abbreviations: *au*, arbitrary units; *CXCL5*, C-X-C Motif Chemokine Ligand 5; *IGFPB2*, Insulin Like Growth Factor Binding Protein 2; *RBP4*, Retinol Binding Protein 4; *REG3G*, Regenerating islet-derived protein 3 gamma.

**Extended Data Fig. 8 |:**
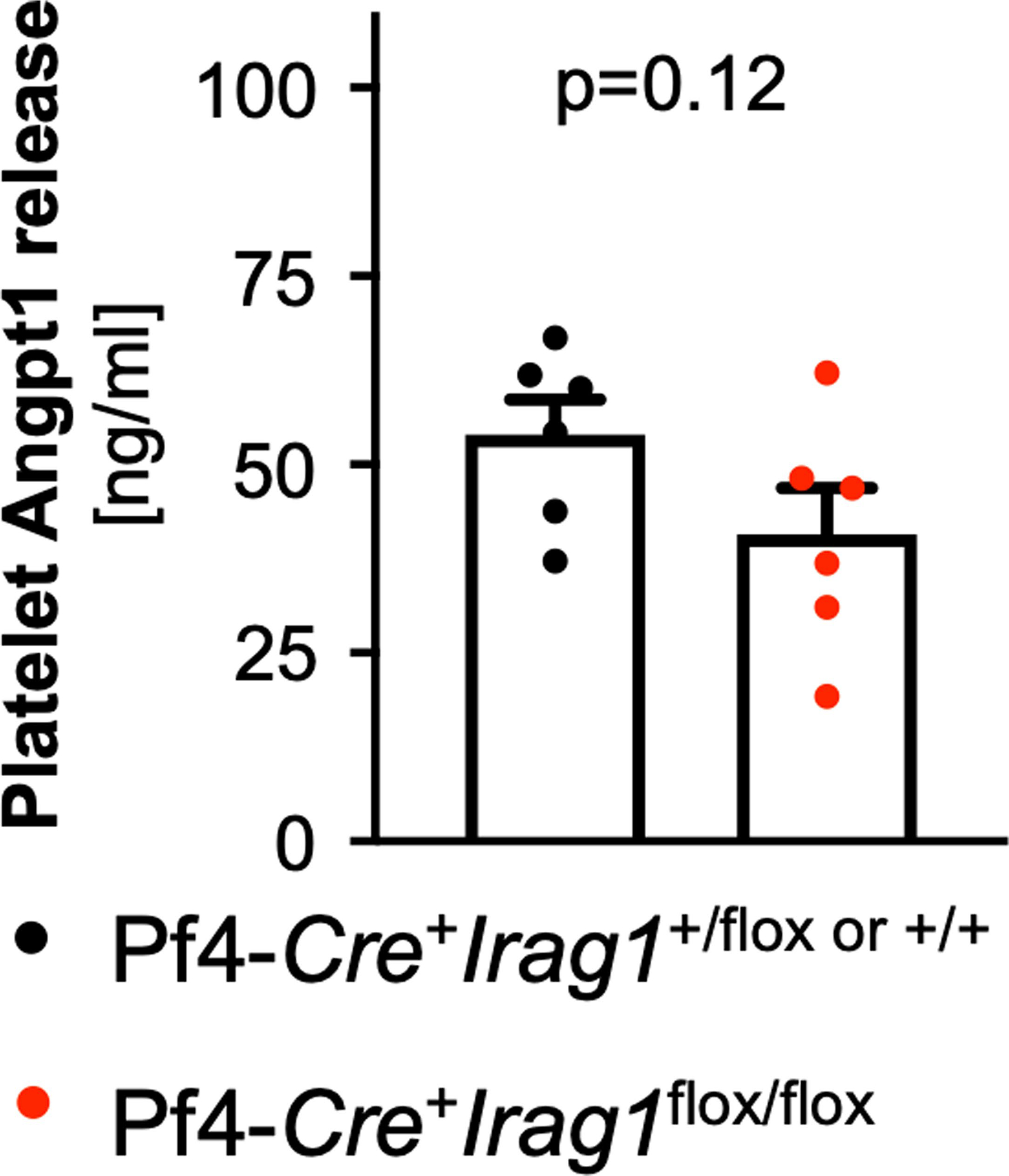
ngiopoietin-1 (ANGPT1) release by control or Pf4-*Cre*^+^*Irag1*^*LoxP/LoxP*^ platelets after activation by shaking. **A** Each symbol represents one independent animal (n = 6). Two-sided unpaired t-test. Data are mean and s.e.m.

**Fig. 3 |:**
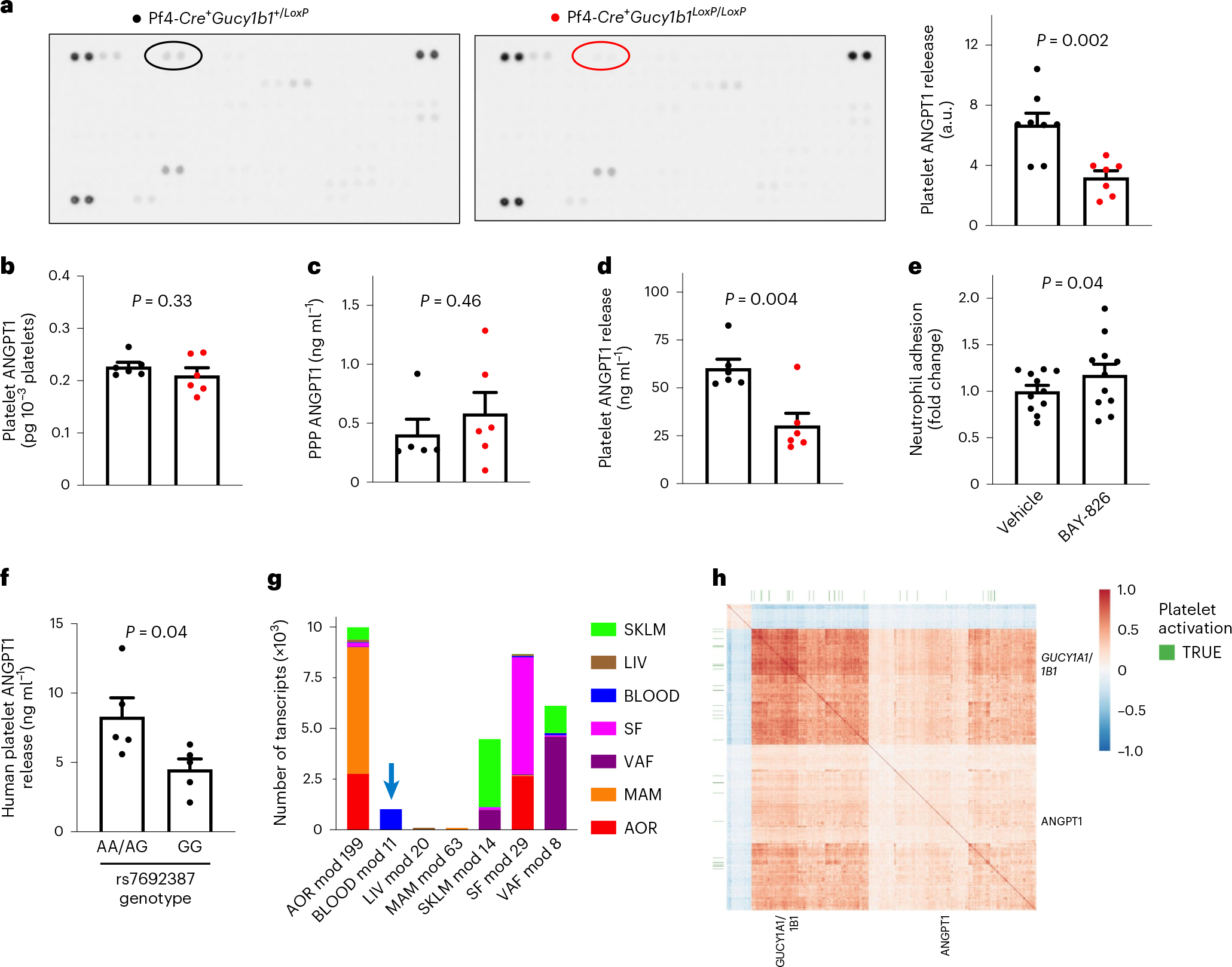
Platelet sGC influences the release of ANGPT1. **a**, Left, identification of ANGPT1 (encircled) as differentially released protein from activated Pf4-*Cre*^+^*Gucy1b1*^+/*LoxP*^ and Pf4-*Cre*^+^*Gucy1b1*^*LoxP*/*LoxP*^ platelets. Right, quantification of ANGPT1 signal in 8 Pf4-*Cre*^+^*Gucy1b1*^+/*LoxP*^ and 7 Pf4-*Cre*^+^*Gucy1b1*^*LoxP*/*LoxP*^ mice. Two-sided unpaired *t*-test. **b**–**d**, Quantification of platelet ANGPT1 content (*n* = 6 independent animals) (**b**), PPP ANGPT1 (*n* = 5 and 6 independent animals, respectively) (**c**) and released ANGPT1 as determined in 6 independent animals per group by ELISA (**d**). Two-sided unpaired *t*-test. **e**, WT neutrophil adhesion to WT ECs after incubation with supernatant of activated WT platelets in the absence and presence of the Tie2 inhibitor BAY-826 (0.5 μM). Two-sided paired *t*-test on *n* = 11 sample pairs derived from independent animals. **f**, Platelet ANGPT1 release in five humans carrying the *GUCY1A1* (rs7692387) non-risk (AA, AG genotype) allele and five homozygous carriers of the risk allele (GG genotype). Each symbol represents one individual. Two-sided unpaired *t*-test. Data are the mean ± s.e.m. **g**, STARNET coexpression modules containing *ANGPT1* from multitissue RNA-seq sampling of approximately 600 patients with CAD. The arrow denotes coexpression module 11 from whole-blood samples (BLOOD). **h**, Heatmap of Pearson’s correlation coefficients of genes in coexpression module 11, showing positive correlation of *ANGPT1*, *GUCY1A1* and *GUCY1B1* along with enrichment for platelet activation genes (false discovery rate = 6.863 × 10^−13^, Enrichr, KEGG pathway). AOR, aorta; LIV, liver; MAM, mammary artery; SF, subcutaneous fat; SKLM, skeletal muscle; VAF, visceral fat.

We observed the influence of a soluble factor released by platelets on leukocyte adhesion to ECs. To identify such factors, we next performed cytokine profiling with supernatant of activated Pf4-*Cre*^+^*Gucy1b1*^*LoxP*/*LoxP*^ and Pf4-*Cre*^+^*Gucy1b1*^+/*LoxP*^ platelets. Signal intensity analysis revealed lower angiopoietin-1 (ANGPT1) levels in the supernatant from Pf4-*Cre*^+^*Gucy1b1*^*LoxP*/*LoxP*^ platelets (3.2 ± 0.4 (*n* = 7) versus 6.7 ± 0.8 (*n* = 8) arbitrary units (a.u.), *P* = 0.002; Fig. 3a and Extended Data Fig. 7). We next aimed at replicating this finding in an independent cohort of mice using an enzyme-linked immunosorbent assay (ELISA). Importantly, ANGPT1 levels were comparable in quiescent platelets (0.21 ± 0.01 versus 0.23 ± 0.01 pg 10^−3^ platelets, *n* = 6 each, *P* = 0.33; Fig. 3b) as well as in platelet-poor plasma (PPP) from Pf4-*Cre*^+^*Gucy1b1*^*LoxP*/*LoxP*^ and Pf4-*Cre*^+^*Gucy1b1*^+/*LoxP*^ mice (0.6 ± 0.2 ng ml^−1^ (*n* = 6) versus 0.4 ± 0.1 ml^−1^ (*n* = 5), *P* = 0.46; Fig. 3c). In line with the explorative analysis displayed in Fig. 3a, we found Pf4-*Cre*^+^*Gucy1b1*^*LoxP*/*LoxP*^ platelets to release reduced amounts of ANGPT1 on activation (30.4 ± 6.4 versus 60.3 ± 4.7 ng ml^−1^, *n* = 6 each, *P* = 0.004; Fig. 3d). ANGPT1 decreases particularly vascular endothelial growth factor (VEGF)-mediated adhesion of leukocytes to ECs^11^ and binds to the Tie2 receptor on ECs^12^. We used the Tie2 inhibitor BAY-826 to investigate whether Tie2 inhibition influences leukocyte adhesion and found a 17% (±1.2%, *n* = 11 experiments, *P* = 0.04) increase in leukocyte adhesion secondary to the inhibition of the ANGPT1 receptor (Fig. 3e). These data demonstrate that ANGPT1 represents a candidate for mediating the effects of platelet sGC on leukocyte recruitment. Of note, inositol 1,4,5-trisphosphate receptor-associated cGMP-kinase substrate (IRAG), which represents a downstream effector of cGMP specifically in modulating platelet activity, is encoded by the *IRAG1* (ref. ^13^) (previously *IRAG* or as human homolog *MRVI1*) gene and has also been associated with CAD by GWAS^14^. To investigate whether differential ANGPT1 release is mediated via IRAG, we generated Pf4-*Cre*^+^*Irag1*^*LoxP*/*LoxP*^ mice and investigated platelet ANGPT1 release compared to the respective controls. In contrast to Pf4-*Cre*^+^ *Gucy1b1*^*LoxP*/*LoxP*^ platelets, we did not detect a difference between the genotypes in this experiment indicating that the influence of sGC on platelet ANGPT1 release is independent of IRAG (Extended Data Fig. 8). It was previously shown that the genotype of the rs7692387 CAD risk allele at the *GUCY1A1* locus^4^ influences *GUCY1A1* expression in different tissues^8^ and sGC α_1_ protein levels in platelets in particular^7^. Therefore, we tested whether the rs7692387 genotype is associated with ANGPT1 release from platelets in healthy human individuals (*n* = 5 each; for characteristics see Supplementary Table 3) and found that homozygous carriers of the CAD risk allele G display lower ANGPT1 release compared to heterozygous or homozygous carriers of the non-risk allele (4.5 ± 0.7 versus 8.3 ± 1.4 ng ml^−1^, *P* = 0.04; Fig. 3f).

To explore the role of ANGPT1 in relation to the genes encoding sGC in humans, we queried the STARNET database which contains bulk RNA sequencing (RNA-seq) data from seven cardiometabolic tissues from patients undergoing coronary artery bypass graft surgery^15,16^. *ANGPT1* was detected per tissue in seven distinct coexpression modules (Fig. 3g). Of note, *ANGPT1* was coexpressed with both *GUCY1A1* and *GUCY1B1* (encoding sGC α_1_ and sGC β_1_) in all tissues except mammary artery represented by coexpression module 63 (Fig. 3g). These data suggest ubiquitous presence and clinical variation in the amounts of platelets. To investigate circulatory rather than tissue-resident platelets, we further analyzed coexpression module 11 from whole-blood samples; this module consisted of 1,016 genes and was estimated to account for 4.9% of CAD heritability by considering expression quantitative trait locus (eQTL) genes in a meta-analysis of nine GWAS using the restricted maximum likelihood method^17^. Coexpression analysis revealed positive correlation between *GUCY1A1*/*GUCY1B1* and *ANGPT1* expression and enrichment for genes involved in the Kyoto Encyclopedia of Genes and Genomes (KEGG) pathway platelet activation (Fig. 3h). Altogether this suggests that a substantial proportion of CAD heritability could be mediated by platelets. Furthermore, Gene Ontology (GO) enrichment analysis of this module (Supplementary Table 4) revealed, among others, ‘response to wounding’ (*P* = 4.71 × 10^−63^), ‘wound healing’ (*P* = 1.84 × 10^−60^), blood coagulation (*P* = 1.99 × 10^−48^) and ‘regulation of locomotion’ (*P* = 6.64 × 10^−44^). These findings support the role of platelets and the interaction of ANGPT1 with sGC in human CAD.

### Modulation of ANGPT1 release by sGC stimulation

**Extended Data Fig. 9 |:**
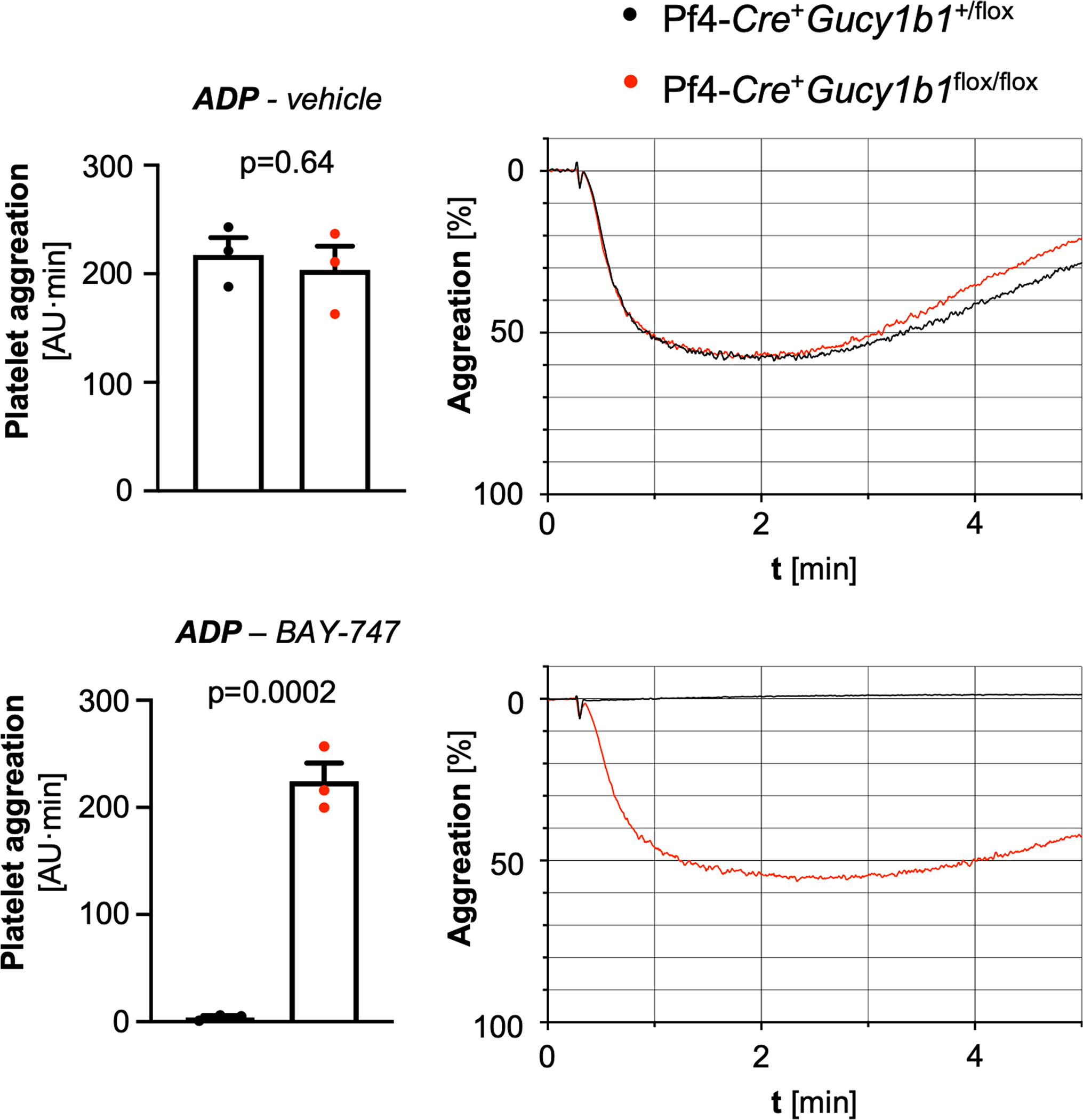
Adenosine diphosphate-induced platelet aggregation in Pf4-*Cre*^+^*Gucy1b1*^+/LoxP^ and Pf4-*Cre*^+^*Gucy1b1*^*LoxP/LoxP*^ platelets after pretreatment with vehicle or BAY-747. Each symbol represents one independent animal (n = 3). Two-sided unpaired t-test. Data are mean and s.e.m. Abbreviation: *ADP*, adenosine diphosphate.

**Fig. 4 |:**
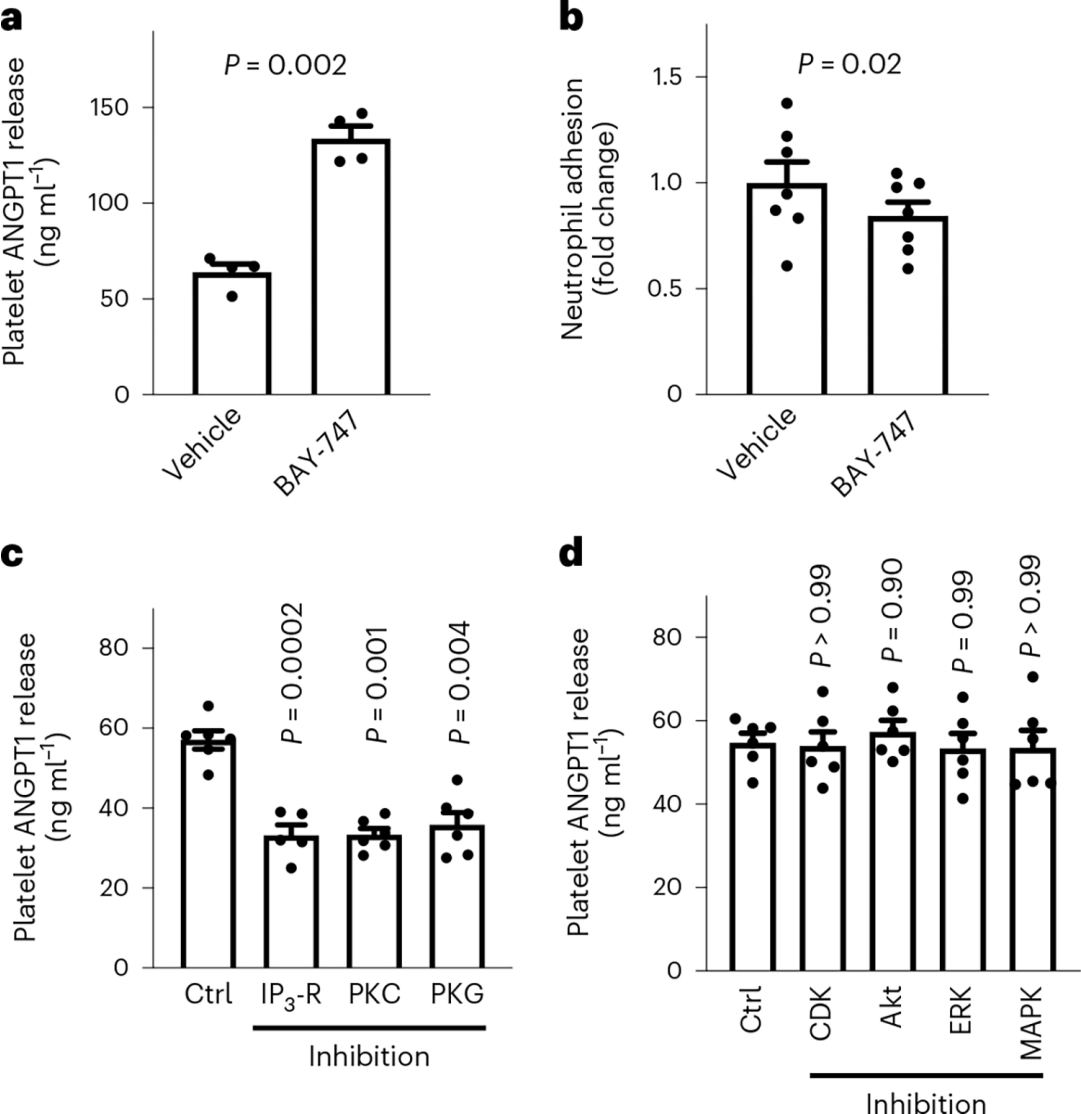
ANGPT1 release from platelets is influenced by sGC via the PKC pathway. **a**, ANGPT1 release by WT platelets incubated with 150 ppm BAY-747 or vehicle (*n* = 4 sample pairs from independent animals). **b**, WT neutrophil adhesion to WT ECs after incubation with supernatant from *n* = 7 activated WT platelets from independent animals that were preincubated with either vehicle or 150 ppm BAY-747. **c**, Inhibition of IP_3_-R (10 μM 2-APB), PKC (5 μM Ro 32–0432), PKG (10 μM KT-5823) and measurement of ANGPT1 release from platelets (*n* = 6). One outlier was removed from the PKG group (*n* = 5) according to the ROUT test. **d**, Inhibition of mitogen-activated protein kinase (MAPK) (10 μM VX-702), cyclin-dependent kinase (CDK) 2/5/9 (100 nM dinaciclib), extracellular signalregulated kinase (ERK) (10 μM ravoxertinib), and Akt kinase (1 μM MK-2206) and measurement of ANGPT1 release from platelets (*n* = 6). Each symbol represents paired samples derived from independent animals. **a**,**b**, Two-sided paired *t*-test. **c**,**d**, Mixed-effects analysis (ANOVA with Dunnett multiple comparison test). Data are the mean ± s.e.m.

The sGC represents a druggable target and sGC stimulators are used in different clinical scenarios, for example, riociguat in pulmonary arterial hypertension and chronic thromboembolic pulmonary hypertension^18^ or vericiguat in chronic heart failure^19^. We next aimed at investigating whether modulation of sGC using a vericiguat-like sGC stimulator (BAY-747) can influence the release of ANGPT1 and vascular inflammation. To this end, we first incubated platelets from WT mice with BAY-747 or vehicle and analyzed ANGPT1 release and leukocyte adhesion to ECs. Of note, the platelets of Pf4-*Cre*^+^*Gucy1b1*^*LoxP*/*LoxP*^ mice are not responsive to BAY-747 regarding, for example, platelet aggregation (Extended Data Fig. 9). Stimulation of sGC with BAY-747 in WT platelets doubled ANGPT1 release (133.8 ± 6.5 ng ml^−1^ versus 64.0 ± 4.3 ng ml^−1^, *n* = 4 each, *P* = 0.002; Fig. 4a) and reduced neutrophil adhesion to ECs by 15.6% (± 6.5%, *n* = 7 each, *P* = 0.02; Fig. 4b). To determine which downstream cGMP pathways instead of IRAG could influence ANGPT1 release on platelet activation by shaking, we performed serine/threonine kinase profiling in WT platelets that were preincubated with BAY-747 and then activated by shaking. As displayed in Supplementary Table 5, sGC stimulation led to a marked increase in the activation of kinases from the AGC group, which include protein kinase C (PKC) and protein kinase G (PKG). To determine whether the PKC pathway is involved in ANGPT1 release and whether canonical cGMP signaling is involved in its regulation, we next inhibited the inositol 1,4,5-trisphosphate receptor (IP_3_-R), PKC and PKG directly and measured ANGPT1 release secondary to activation by shaking (Fig. 4c). Compared to vehicle, inhibition of IP_3_-R (−23.8 ± 2.8 ng ml^−1^, *n* = 5, *P*_adj_ = 0.01), PKC (−23.9 ± 1.4 ng ml^−1^, *n* = 6, *P*_adj_ < 0.001) and PKG (−21.3 ± 3.5 ng ml^−1^, *n* = 6, *P*_adj_ = 0.004) reduced ANGPT1 release. In contrast, inhibition of further downstream pathways did not result in altered ANGPT1 release (Fig. 4d). In an analysis of phosphorylated peptides, main targets of NO–cGMP signaling were detected, including endothelial NO synthase and vasodilatorstimulated phosphoprotein being among the top ten phosphorylated targets (Supplementary Table 6). These data indicate that ANGPT1 release by platelets induced by shaking is mediated via the PKC pathway and modulated by cGMP via canonical cGMP signaling.

### Therapeutic potential of stimulating sGC in atherosclerosis

**Extended Data Fig. 10 |:**
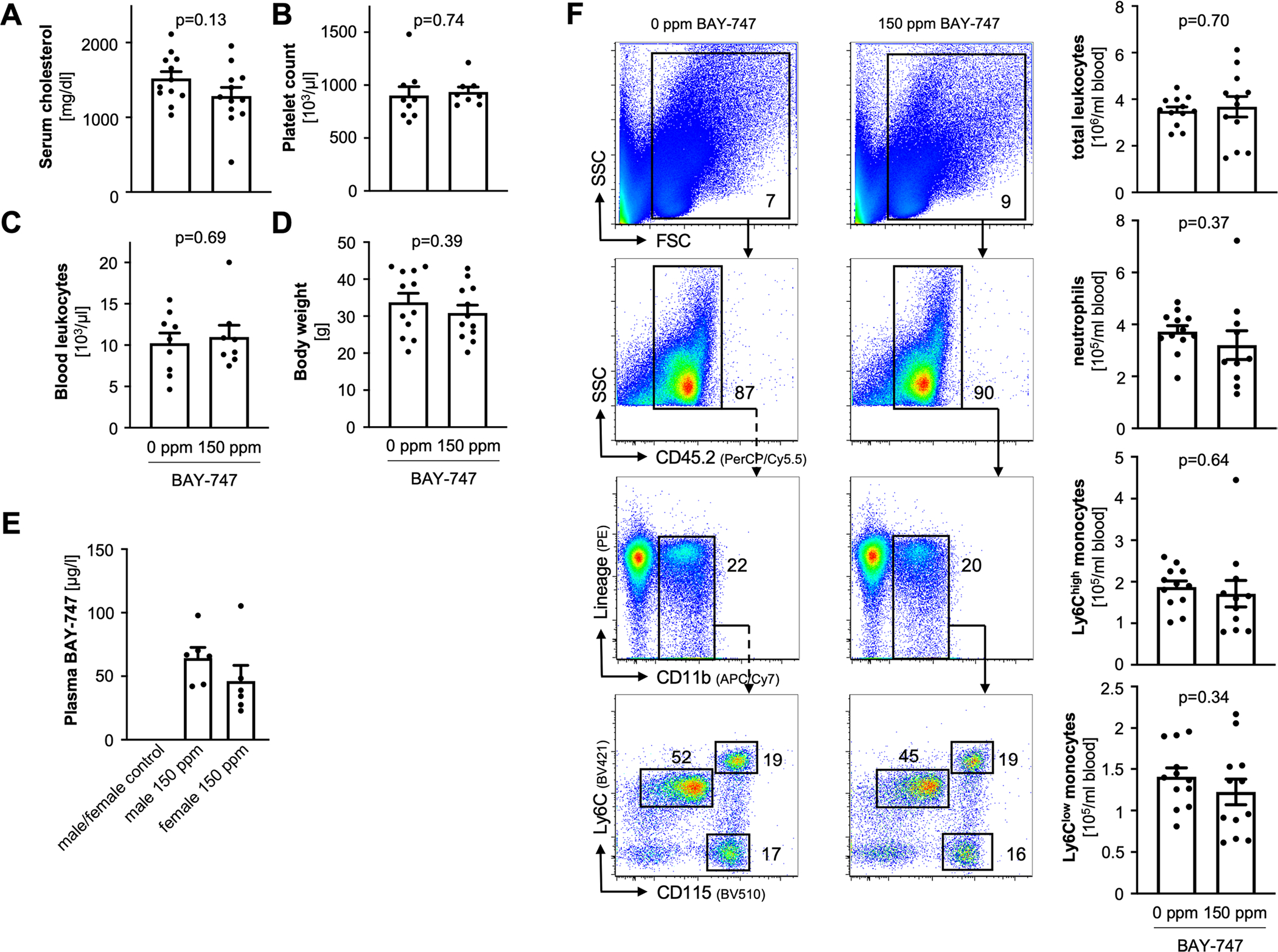
Characteristics of *Ldlr*^−/−^ mice receiving 0 ppm compared to *Ldlr*^−/−^ mice receiving 150 ppm BAY-747 after ten weeks of Western diet. **a**. Serum cholesterol levels (n = 12). **b**. Platelet count (n = 9 in 0 ppm, n = 8 in 150 ppm group). **c**. Blood leukocytes (n = 9 in 0 ppm, n = 8 in 150 ppm group). **d**. Body weight after (n = 12). **e**. Plasma concentrations of the soluble guanylyl cyclase stimulator BAY-747 in treated mice. **f**. Blood leukocyte numbers and subsets. Each symbol represents one independent animal (n = 12). Two-sided unpaired t-test. Two outliers were removed in the analysis of **F**: neutrophils (150 ppm group; n = 10) and Ly6C^high^ monocytes (150 ppm group; n = 11) according to the ROUT method. Data are mean and s.e.m.

**Fig. 5 |:**
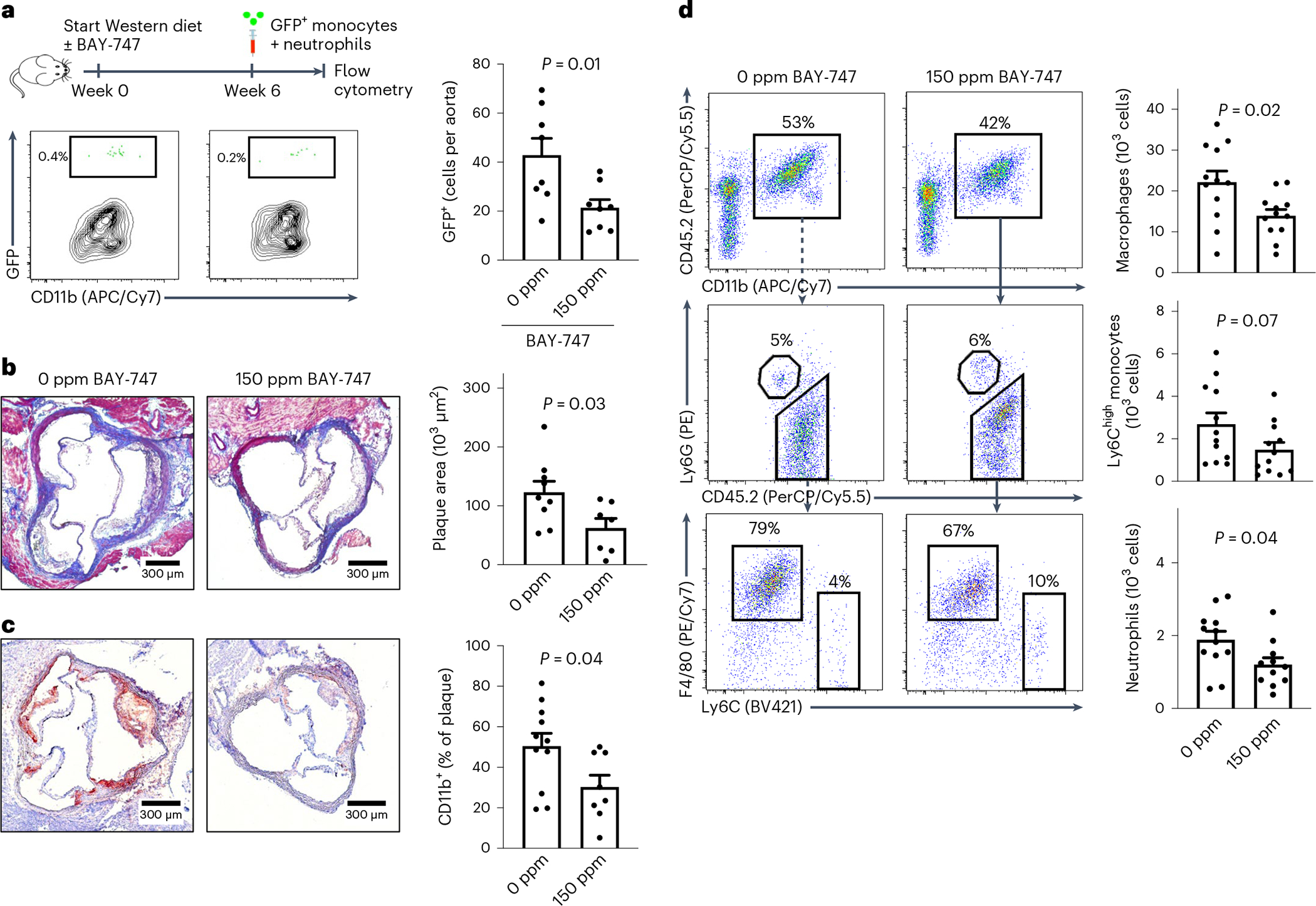
Pharmacological sGC stimulation influences leukocyte recruitment, atherosclerotic plaque formation and vascular inflammation. **a**, Adoptive transfer of GFP^+^ leukocytes: study scheme, flow cytometry plot (left) and quantification (right) of GFP^+^ cells (*n* = 8 independent animals). **b**, Aortic root atherosclerotic plaques in *Ldlr*^−/−^ mice that were fed a Western diet for 10 weeks containing 0 (control group, *n* = 9) or 150 ppm BAY-747 (*n* = 7). **c**, CD11b^+^ area of aortic roots in mice from the control (*n* = 11) and treatment groups (*n* = 8). **d**, Quantification of vascular inflammation by flow cytometry analysis of aortic cell suspensions of mice in the control (*n* = 12) and treatment groups (*n* = 12). Each symbol represents one independent mouse. Two-sided unpaired *t*-test. Data are the mean ± s.e.m. One outlier was removed in **d** in the analysis of neutrophils (150 ppm; *n* = 11) according to the ROUT test.

To investigate whether sGC stimulation in vivo can reduce recruitment from blood to the vascular wall, we fed *Ldlr*^−/−^ mice a Western diet containing 0 or 150 parts per million (ppm) BAY-747 and adoptively transferred green fluorescent protein (GFP)^+^ myeloid cells after 6 weeks. We retrieved GFP^+^ monocytes admixed with neutrophils from naïve transgenic Ubc-GFP mice (all leukocytes express GFP^+^) and injected the cells intravenously into *Ldlr*^−*/*−^ mice (all cells are GFP^−^). After a further 24 h, mice were killed and the numbers of GFP^+^ cells were determined using flow cytometry of aortic cell suspensions. Mice that received the Western diet containing the sGC stimulator displayed reduced numbers of aortic GFP^+^ cells (21.4 ± 3.3 versus 42.8 ± 6.9 cells, *n* = 8 each, *P* = 0.01; Fig. 5a). These data and the data displayed in Fig. 4 indicate that pharmacological sGC stimulation can modulate platelet ANGPT1 release, in vitro leukocyte adhesion, and in vivo leukocyte recruitment. To determine whether such treatment can reduce atherosclerotic plaque formation, we again fed atherosclerosis-prone *Ldlr*^−/−^ mice a Western diet for ten weeks. In one group, the diet contained 150 ppm BAY-747 (treatment group) while the other group received a Western diet without (0 ppm) BAY-747 (control group). Both groups had elevated serum cholesterol levels without significant difference between the two groups; similarly, platelet count, blood leukocytes and body weight after the diet were comparable (Extended Data Fig. 10a–d). Under steady-state conditions, BAY-747 plasma levels in male and female mice receiving BAY-747 were 61.7 ± 1.4 μg l^−1^ and 40.3 ± 1.7 μg l^−1^ (*n* = 6, each), respectively; in the control group, BAY-747 was not detectable in plasma as expected (Extended Data Fig. 10e). In the aortic root, we found significantly reduced atherosclerotic plaque formation in mice of the treatment group (62.5 ± 16.1 μm^2^ (*n* = 7) versus 123.1 ± 18.6 μm^2^ (*n* = 9), *P* = 0.03; Fig. 5b). Furthermore, we detected fewer leukocytes in the aortic roots of those animals (30.3 ± 5.8% (*n* = 8) versus 50.5 ± 6.3% (*n* = 11) of plaque area, *P* = 0.04; Fig. 5c). In cell suspensions of the whole aorta, we found fewer numerous macrophages (13,915 ± 1,550 versus 22,156 ± 2,737, *n* = 12 each, *P* = 0.02; Fig. 5d), Ly6C^high^ monocytes (1,485 ± 348 versus 2,688 ± 531, *n* = 12 each, *P* = 0.07; Fig. 5d) and neutrophils (1,201 ± 192 (*n* = 11) versus 1,885 ± 232 (*n* = 12), *P* = 0.04; Fig. 5d) per aorta in mice treated with the sGC stimulator compared to mice receiving the control diet. Blood leukocyte numbers as determined by flow cytometry and leukocyte subsets were comparable between both groups (Extended Data Fig. 10f). Taken together, these data indicate that pharmacological sGC stimulation reduces atherosclerotic plaque formation and vascular inflammation via dampened recruitment of inflammatory leukocytes from blood to plaque.

## Discussion

NO–sGC–cGMP signaling has important functions in several cell types. For instance, increasing intracellular cGMP levels inhibits migration of vascular smooth muscle cells and aggregation of platelets, respectively^20,21^. The observation that genes encoding key proteins in this pathway were associated with CAD and premature MI by GWAS^4,14,22,23^ and exome sequencing studies^5,8,24^ renders an important role in the pathophysiology of coronary atherosclerosis likely. In this study, we sought to specifically investigate the role of platelet sGC in atherosclerosis because we found that carriers of the common, noncoding risk variant rs7692387 identified by GWAS^4^ displayed reduced expression of sGC in platelets and, as a consequence, reduced inhibition of platelet aggregation on NO stimulation^7^. In addition, we observed that in contrast to the general population, individuals who are homozygous for this variant had a benefit from aspirin treatment in primary prevention of cardiovascular events^9^. In a series of in vivo and in vitro experiments, we observed larger atherosclerotic plaques in the aortic roots of mice lacking platelet sGC compared to sGC WT mice. Platelet sGC has just recently been described to act as an endogenous brake on platelet aggregation^25^. Indeed, it has been hypothesized that platelets adhering to endothelial or plaque erosions may be activated more or less depending of the availability of sGC. This may stimulate inflammation locally and, subsequently, facilitate atherosclerotic plaque formation. While this is a hypothesis, we report evidence that platelet sGC influences the release of the soluble factor ANGPT1 with reduced amounts released by platelets lacking sGC. The role of ANGPT1 in atherosclerosis is controversial since there are reports describing a protective^11,12,26^ and a deleterious role^27^. However, given the cellular findings describing the inhibitory effect of ANGPT1 on leukocyte adhesion and vascular atherosclerosis reported in the literature^11^, which were confirmed in this study, and the beneficial role of ANGPT1–Tie2 signaling in inflammatory diseases in general^28^, it represents an interesting candidate mediating the downstream effects of platelet sGC signaling. In addition, its effects might be depending on coreleased factors. Importantly, the role of sGC in platelets exceeds the role as a brake on aggregation: while sGC passivates platelet aggregation, it enhances the release of ANGPT1 when platelets are modestly activated. The notion that this effect is independent of aggregation is further supported by the findings that (1) IRAG, the mediator of cGMP-dependent inhibition of platelet aggregation^29^ was not significantly involved in ANGPT1 release and (2) that ANGPT1 release was induced by shaking but not, for example, ADP or arachidonic acid. ANGPT1 release rather seems to be mediated by PKC activity and modulated via canonical cGMP signaling. It is important to acknowledge that the content of ANGPT1 in WT and knockout platelets was similar further indicating a direct link between sGC availability and ANGPT1 release. This is further emphasized by the finding that a genetically determined reduction, but not lack of, sGC in the platelets of carriers of the CAD-associated risk variant rs7692387 was associated with a reduction in ANGPT1 release. This, on the one hand, supports the translational relevance of this by nature artificial in vitro and animal studies; on the other hand, it raises the question whether modulators of sGC function could be used to prevent and treat coronary atherosclerosis. Indeed, stimulators of sGC are emerging as therapeutic compounds for different cardiovascular diseases (for an overview see Sandner et al.^30^). Recently, the sGC stimulator vericiguat was found to lower the risk of death from cardiovascular causes or hospitalization for heart failure in patients with heart failure with reduced ejection fraction^19^. However, data on atherosclerotic plaque formation and ischemic cardiovascular events are lacking thus far.

To this end, we investigated whether sGC stimulation using a vericiguat-like stimulator, BAY-747, can modulate the reported cellular and phenotypic effects. First, we found that sGC stimulation can increase ANGPT1 release and subsequently reduce leukocyte adhesion to ECs in vitro. Since we postulate that sGC contributes to reducing vascular inflammation, we next studied whether sGC stimulation can alter the recruitment of inflammatory leukocytes from blood to plaque. In an adoptive transfer experiment, we observed a reduction in leukocyte recruitment that is furthermore in line with previous reports showing anti-inflammatory effects of cGMP-increasing pharmacological compounds^31,32^. The finding of multiple genome-wide significant hits in genes that encode proteins tightly involved in the formation (*NOS3* (ref. ^22^), *GUCY1A1* (ref. ^4^)), fate (*PDE5A*^23,24^), or mediating the downstream effects of cGMP (*IRAG1* (ref. ^14^), *PDE3A*^33^) and the observation that sGC levels are reduced in atherosclerotic tissues^34^ increase the likelihood that targeting sGC might be beneficial in CAD. In atherosclerosis-prone mice on a Western diet, stimulating sGC with BAY-747 led to a reduction in atherosclerotic plaque formation and vascular inflammation warranting further investigation into this promising pharmacological treatment strategy. Further evidence might be taken from a recent study that compared two pharmacological strategies which are used to treat erectile dysfunction: compared to alprostadil, that is, prostaglandin E1, treatment with the inhibitor of phosphodiesterase 5A, sildenafil, was associated with a reduced risk of all-cause mortality and MI in men suffering from CAD^35^. However, to definitively prove a beneficial role in CAD, prospective clinical trial data are needed.

**Fig. 6 |:**
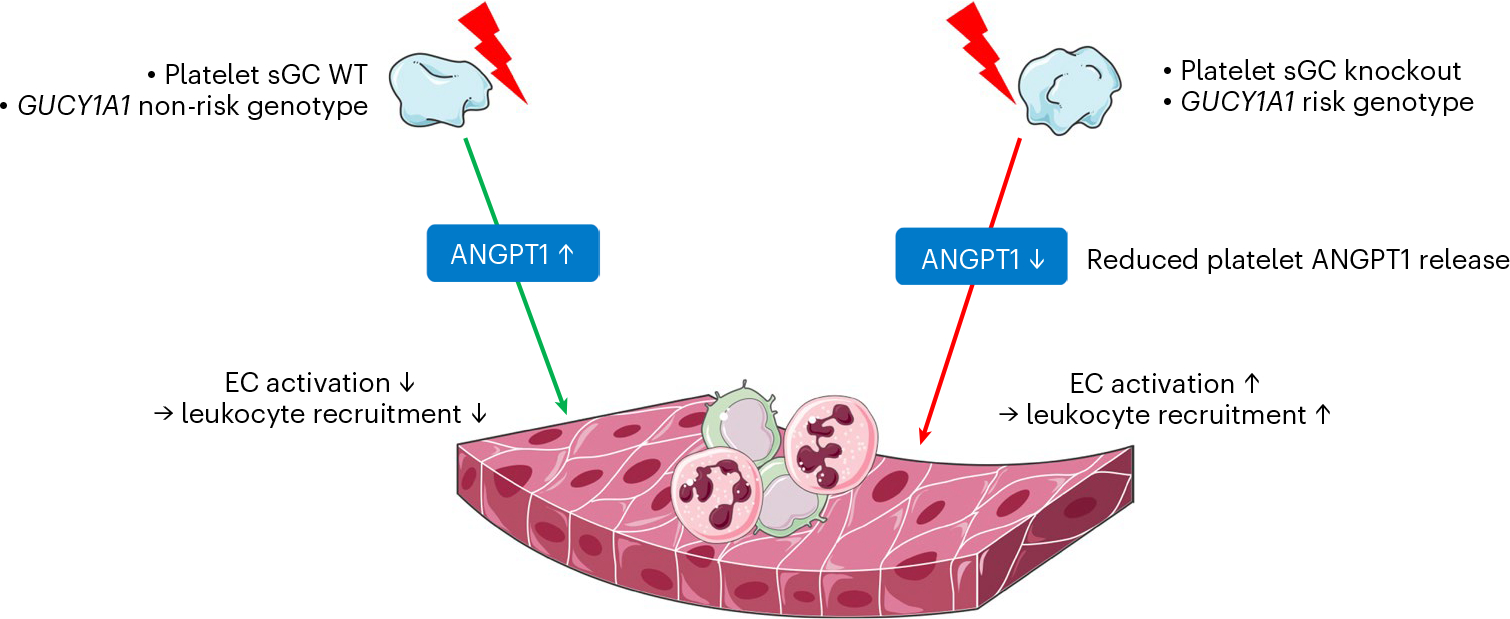
In the event of platelet activation, for example, by shear stress, sGC counterbalances proinflammatory activation of ECs, for example, by release of ANGPT1. If sGC levels are reduced, for example, in the mouse model used in this study or in platelets of homozygous carriers of the CAD-associated risk variant, less ANGPT1 is released. Subsequently enhanced EC activation and leukocyte recruitment contribute to atherosclerotic plaque formation. This figure contains modified image material available at Servier Medical Art under a Creative Commons Attribution 3.0 Unported License.

Taken together, we provide further evidence for a crucial role of platelets in atherosclerosis in general, and of platelet sGC in particular. As shown by our in vitro studies using human and murine biospecimen and in vivo studies, we postulate an endogenous inhibitory role of platelet sGC on EC-mediated leukocyte recruitment (Fig. 6). We are aware that platelets are not the only cell type in which sGC activity and genetic variants modulating its availability influence CAD risk. Modulating sGC activity, especially using stimulators, might nevertheless be a promising therapeutic strategy exceeding the effects on platelet sGC.

Our study has several limitations. First, this is an in vitro and mouse in vivo study that cannot resemble the human physiology and pathophysiology. However, the finding that lack of platelet sGC in mice and genetically determined reduced platelet sGC α_1_ in humans both reduce release of platelet ANGPT1 supports a possible translation. Furthermore, similar to humans, a genetically determined reduction in *Gucy1a1* messenger RNA was associated with increased atherosclerotic plaque formation in the hybrid mouse diversity panel^7,36^. Second, although we have shown that reduced ANGPT1 release and enhanced leukocyte adhesion to ECs is linked to reduced or lacking platelet sGC availability and that stimulation of sGC can modulate these downstream effects, the exact molecular mechanism linking sGC and ANGPT1 release is to be explored. While it is well known that platelets contain at least three types of secretory granules, with α-granules that also harbor ANGPT1 (ref. ^37^) being the most abundant type, there is also evidence for the existence of functionally distinct subpopulations within α-granules^38^, which may allow selective release of their contents by different stimuli^39,40^. A similar observation has been reported in neutrophils regarding storage of ANGPT1 and VEGF^41^. We further provide evidence that it is independent of IRAG but mediated via canonical cGMP signaling. Third, we know that ANGPT1 is likely not the only mediator of sGC effects on atherosclerosis. Rather, ANGPT1 might modulate the effect of other cytokines and mediators that are released by platelets on modest activation. Fourth, although we show that platelet sGC activity influences leukocyte recruitment and atherosclerotic plaque formation, the benefit of systemic sGC stimulators might be influenced by the effects on sGC in other cell types, for example, vascular smooth muscle cells. Lastly, we cannot generalize a benefit of sGC stimulation to human platelets. Yet our study can, in addition to its biological implications, be regarded as hypothesis-generating for future clinical trials investigating whether modulating cGMP formation is useful in addition to reduce, for example, low-density lipoprotein cholesterol levels and residual inflammatory risk.

## Methods

### Mouse studies

Animal experiments were conducted in accordance with the German legislation on the protection of animals and approved by the local animal care committee (District Government of Upper Bavaria, GZ: ROB-55.2–2532.Vet_02–15-176). A knockout of platelet sGC can be reached via deleting the sGC α_1_ or β_1_ subunit. Both subunits form a functional unit so a knockout can be generated by knocking out either subunit. Since the knockout of sGC β_1_ has been established in cell-specific models^42,43^, we chose to study sGC β_1_ knockout mice although the gene encoding sGC α_1_ was primarily identified as a CAD risk gene^4,5^. Platelet-specific sGC knockout mice (Pf4-*Cre*^+^*Gucy1b1*^*LoxP*/*LoxP*^) were obtained by crossing conditional NO-GC β1 knockout mice (*Gucy1b1*^*LoxP*/*LoxP*^)^44^ and Pf4-*Cre*^*tg*/+^ mice (Pf4-*Cre*^+^) as described previously^45^. Subsequently, Pf4-*Cre*^+^*Guc y1b1*^*LoxP*/*LoxP*^ mice were crossbred with *Ldlr*^−*/*−^ mice (B6.129S7-*Ldlr*^tm1Her^/J) to induce an *Ldlr*^−*/*−^ pro-atherosclerotic background. For experiments, Pf4-*Cre*^+^*Gucy1b1*^*LoxP*/*LoxP*^*Ldlr*^−/−^ and *Gucy1b1*^+/*LoxP*^*Ldlr*^−/−^ mice were mated to receive control (Pf4-*Cre*^+^*Gucy1b1*^+/*LoxP*^*Ldlr*^−/−^) and platelet-specific sGC knockout (Pf4-*Cre*^+^*Gucy1b1*^*LoxP*/*LoxP*^*Ldlr*^−/−^) littermates. *Irag1*^*LoxP*^/^*LoxP*^ mice^46^ were mated with Pf4-*Cre*^+^ mice and obtained Pf4-Cre^+^*Irag1*^*LoxP*/*LoxP*^ mice were backcrossed to a C57BL/6J background. Pf4-*Cre*^+^*Irag1*^+/*LoxP*^ and Pf4-*Cre*^+^*Irag1*^+/+^ littermates were used as controls. *Ldlr*^−*/*−^, C57BL/6J (WT) and *Ubc-GFP* (C57BL/6-Tg(UBC-GFP)30Scha/J) mice were purchased from The Jackson Laboratory. Only male or male and female mice at a 1:1 ratio at 8–12 weeks of age were used for all experiments. Mice were kept in a specific pathogen-free area with HEPA-filtered room air. Cages were illuminated with an automatic light regime of 12 h in a day–night rhythm and temperature was kept constant between 20 and 22 °C at a humidity of 45–60%.

To compare aor tic plaque sizes between Pf4-*Cre*^+^*Gucy1b1*^*LoxP*/*LoxP*^*Ldlr*^−/−^ and Pf4-*Cre*^+^*Gucy1b1*^+/*LoxP*^*Ldlr*^−/−^ mice, animals were fed a Western diet (21.2% fat and 0.2% cholesterol by weight; TD.88137; Envigo) for 10 weeks. For intravital fluorescence microscopy, Pf4-*Cre*^+^*Gucy1b1*^*LoxP*/*LoxP*^*Ldlr*^−/−^ and Pf4-*Cre*^+^*Gucy1b1*^+/*LoxP*^*Ldlr*^−/−^ mice were fed a Western diet (21.2% fat and 0.2% cholesterol by weight; TD.88137) for 6 weeks. To compare aortic plaque sizes after pharmacological sGC stimulation, *Ldlr*^−*/*−^ mice were fed a Western diet (21.1% crude fat and 0.15% cholesterol, E15721–34; TD.88137 modified; ssniff, Soest) containing either 0 (control group) or 150 ppm (treatment group) of the sGC stimulator BAY-747 (N-(2-amino-2-methylbutyl)-8-((2,6-difluorobenzyl)oxy)-2,6-dimethylimidazo(1,2-a]pyridine-3-carboxamide) (Bayer AG) ad libitum for 10 weeks. For adoptive transfer experiments, *Ldlr*^−*/*−^ mice were fed the same diet for six weeks.

### Human samples

The study protocol was approved by the local ethics committee of the Technical University of Munich (no. 387/17S). Blood was collected from healthy volunteers after signing the informed consent. To determine the genotype of the individuals, DNA was isolated from whole blood using the Puregene Blood Kit (catalog no. 158489; QIAGEN) according to the manufacturer’s protocol. Samples were genotyped for the *GUCY1A1* risk variant using an rs7692387 TaqMan Genotyping Assay (C__29125113_10) on a Viia7 system (both Thermo Fisher Scientific).

### Histology, immunohistochemistry and en face staining

Aortic roots were embedded in optimal cutting temperature compound (catalog no. 62550; Sakura Finetek) and snap-frozen to −80 °C. Frozen samples were cut into 5-μm sections and applied to microscope slides. From the onset of aortic valves, every fifth slide was subjected to tissue staining. For the Masson’s trichrome stain, the procedure of Masson as modified by Lillie was applied according to the manufacturer’s instructions (Sigma-Aldrich). In brief, frozen sections were hydrated and fixated in 4% paraformaldehyde before mordanting in Bouin’s solution (catalog no. HT10132; Sigma-Aldrich) at 56 °C for 15 min. Afterwards, cell nuclei were stained in Weigert’s iron hematoxylin solution (catalog no. HT1079; Sigma-Aldrich) and darkened in running tap water. Specimens were successively subjected to Biebrich scarlet-acid fuchsin solution, phosphotungstic/phosphomolybdic acid solution and aniline blue solution (catalog no. HT15; Sigma-Aldrich) for staining of cytoplasm, muscle and collagen structures, respectively. After rinsing in 1% acetic acid, slides were dehydrated in an increasing ethanol row followed by xylene and covered with mounting medium. Mean total plaque size (in μm^2^) was evaluated for sections showing at least two complete cusps by manually selecting the plaque area in ImageJ2 (version 2.3.0). For immunohistochemistry, specimens were fixed in ice-cold acetone, blocked in 10% rabbit serum (catalog no. S5000; Vector Laboratories) in PBS with Tween-20 (0.2%) and stained in anti-CD11b antibody (1:200, catalog no. 101202; BioLegend) or antimonocyte + macrophage (MOMA) antibody (1:50, catalog no. ab33451; Abcam) overnight, followed by incubations in horseradish peroxidase (HRP)-conjugated rabbit anti-rat secondary antibody (1:200, catalog no. ab6734; Abcam) and AEC substrate (catalog no. ab64252; Abcam). The primary antibodies have been validated by the manufacturers for use in immunohistochemistry on frozen mouse samples. Subsequently, cell nuclei were counterstained with Gill’s hematoxylin solution II (catalog no. 1051752500; Merck Millipore). CD11b or monocyte and macrophage content was quantified as CD11b- or MOMA2-positive area, respectively, per total plaque area by means of automated color thresholding in ImageJ2. For en face analyses, aortae were dissected from the heart to the iliac bifurcation, cleaned of surrounding tissue and fixed for 24 h at 4 °C in a 4% solution of paraformaldehyde in PBS. Samples were washed in 60% isopropanol and incubated for 30 min at 37 °C in a freshly filtered solution of 3 mg ml^−1^ Oil Red O (ORO) (catalog no. O0625; Sigma-Aldrich) in 60% isopropanol. After washing off excess dye in 60% isopropanol, aortae were opened longitudinally, pinned on a black pad and imaged on a Stemi 2000-C microscope with an Axiocam ERc 5 s camera using the ZEN 2.3 blue software (version 2.3.69.1000, Carl Zeiss). The percentage of the lesion area was determined manually as the ORO-positive area of total en face aortic area from aortic root to branch of the right renal artery using ImageJ2. All staining and analyses were performed in blinded fashion.

### Isolation and culture of bone marrow megakaryocytes

Bones of Pf4-*Cre*^+^*Gucy1b1*^+/*LoxP*^ and Pf4-*Cre*^+^*Gucy1b1*^*LoxP*/*LoxP*^ mice were centrifuged at 2,500*g* for 1 min after removing proximal epiphyses^47^. The obtained bone marrow was subjected to 1× RBC lysis buffer (catalog no. 420301; BioLegend), filtered through 70-μm mini-cell strainers and resuspended in IMDM medium (catalog no. 31980030; Thermo Fisher Scientific) supplemented with 10% FCS (catalog no. S0615; Sigma-Aldrich), penicillin-streptomycin (1:100, catalog no. 15070063; Thermo Fisher Scientific), thrombopoietin (200 ng ml^−1^, catalog no. 130–096-301; Miltenyi Biotec) and stem cell factor (20 ng ml^−1^, catalog no. 130–101-693; Miltenyi Biotec) to a concentration of 1 × 10^7^ cells per ml. Cells were cultivated in a humidified incubator with 5% CO_2_ at 37 °C for 9 d and supplemented with fresh medium every third day.

Megakaryocytes were collected by performing two rounds of bovine serum albumin (BSA) density gradient filtration^48^. Briefly, cells were resuspended in PBS and placed on top of two layers of a 1.5 and 3% BSA solution and incubated for 40 min. Sedimented cells were subjected to a second round of density gradient filtration, obtained purified megakaryocytes resuspended in 500 μl of TRIzol (catalog no. 15596026; Thermo Fisher Scientific) and stored at −80 °C for further processing.

### Isolation of nucleic acids and (quantitative) PCR

After adding chloroform, samples were shaken vigorously and centrifuged at 12,000*g* for 15 min and 4 °C. The upper phase containing RNA was further processed using the RNeasy Mini Kit (catalog no. 74139; QIAGEN) according to the manufacturer’s recommendations. RNA was quantified using a NanoQuant Plate on an Infinite M200 PRO plate reader (TECAN) and RNA integrity was measured on a 2100 Bioanalyzer (Agilent Technologies).

After the RNA was transcribed into complementary DNA using the High-Capacity RNA-to-cDNA kit (catalog no. 4388950; Applied Biosystems), real-time quantitative PCR (qPCR) was performed using the TaqMan Fast Universal PCR Master Mix (catalog no. 4366072) and TaqMan probes (*Gucy1a1*, Mm01220285_m1; *Gucy1b1*, Mm00516926_m1; *Gucy1a2*, Mm01253540_m1; *Gucy1b2*, Mm00555742_m1; *Vcam1*, Mm01320970_m1; *Gapdh*, Mm99999915_g1; all Thermo Fisher Scientific). Reactions were performed in a total volume of 10 μl on a ViiA 7 system (Thermo Fisher Scientific). *Gapdh* was used as a housekeeping gene and data were evaluated by conversion to ΔCt values.

### Protein extraction

Lung and aorta were collected from Pf4-*Cre*^+^*Gucy1b1*^+/*LoxP*^ and Pf4-*Cre*^+^*Gucy1b1*^*LoxP*/*LoxP*^ mice after perfusion of organs with PBS and snapfrozen in liquid nitrogen. For protein isolation, specimens were placed in ice-cold radioimmunoprecipitation assay (RIPA) buffer (catalog no. 9806S; Cell Signaling Technology) supplemented with 1:100 protease inhibitor cocktail (catalog no. 1861278; Thermo Fisher Scientific) and disrupted using an electric tissue homogenizer on ice. To isolate peripheral blood mononuclear cells (PBMCs), heparinized full blood was applied onto a layer of Ficoll-Paque Premium (catalog no. 17-5442-02; GE Healthcare) and centrifuged at 400*g* for 30 min without break. The interface containing PBMCs was transferred to new tubes, washed and incubated in RBC lysis buffer (BioLegend) for 5 min. Cells were resuspended in RIPA supplemented with 1:100 protease inhibitor cocktail. For the generation of thrombocyte lysates, platelets were collected from heparinized full blood as stated previously and resuspended in RIPA buffer supplemented with protease inhibitor to a number of 2 × 10^8^ cells per ml. Cells were disrupted by intermittent sonication 3 times for 30 s in an ice bath. Protein concentrations were determined using a bicinchoninic acid assay (catalog no. 23227; Thermo Fisher Scientific) according to the manufacturer’s protocol.

### Immunoblotting

Samples were supplemented with 4× Laemmli buffer (catalog no. 1610747; Bio-Rad Laboratories) containing 355 mM 2-mercaptoethanol and denatured for 5 min at 95 °C. Proteins were separated by SDS–gel electrophoresis using 4–20% Mini-PROTEAN TGX precast gradient gels (catalog no. 4561094; Bio-Rad Laboratories) at 100 V for 1.5 h in 1× Tris/glycine/SDS buffer (catalog no. 161–0772; Bio-Rad Laboratories). Wet blotting (25 mM Tris, 192 mM glycine, 20% v/v methanol, pH 8.3) was performed at 100 V for 90 min using methanol-activated polyvinylidene difluoride membranes. Membranes were blocked for 1 h at room temperature in PBS-T (PBS with 0.2% Tween) containing 5% nonfat dry milk powder (NFDM, catalog no. A0830; AppliChem). Primary antibodies (anti-mβ1, directed against the β_1_ subunit of the sGC^44^ diluted 1:1,000 and GAPDH, catalog no. 8884S; Cell Signaling Technology, 1:200,000 in 2.5% NFDM-PBS-T) were incubated at 4 °C overnight, followed by a 1-h incubation at room temperature with anti-rabbit HRP-conjugated secondary antibody (1:100,000 in 2.5% NFDM-PBS-T, catalog no. 7074; Cell Signaling Technology). For signal detection, membranes were developed with SuperSignal West Dura Extended Duration Substrate (catalog no. 34075; Thermo Fisher Scientific) according to the manufactureŕs recommendations and signal intensities were detected using an ImageQuant 800 imager (GE Healthcare).

### Platelet aggregation

Platelet-rich plasma (PRP) was collected from Pf4-*Cre*^+^*Gucy1b1*^+/*LoxP*^ and Pf4-*Cre*^+^*Gucy1b1*^*LoxP*/*LoxP*^ mice as stated in the manuscript and thrombocyte count was measured using an automated hematology analyzer (XP-300; Sysmex Corp). PRP was centrifuged at 700*g* for 10 min to receive PPP used for blanking. Samples were incubated at 37 °C in glass cuvettes with constant stirring on an 8-channel personal computer-controlled platelet aggregation profiler (PAP-8, Biodata Corp) either in the presence of sodium nitroprusside (final concentration 10 μmol l^−1^, catalog no. HN34.1; Carl Roth), BAY-747 (150 ppm) or vehicle (dimethyl sulfoxide (DMSO), 0.4%) for 2 min. Subsequently, thrombocyte aggregation was induced by the addition of ADP (final concentration 2 μmol l^−1^, catalog no. 0203001; mölab), Ala-Tyr-Pro-Gly-Lys-Phe-NH_2_ (final concentration 75 μM, catalog no. A3227; Sigma-Aldrich), U46619 (final concentration 5 μM, catalog no. 1932; Tocris Bioscience) and collagen (final concentration 1 μg ml^−1^, catalog no. 0203009). Platelet aggregation was recorded over 5 min to measure the area under the curve and displayed as a.u. min.

### Generation of supernatant from activated platelets

A total of 800 μl each of blood from Pf4-*Cre*^+^*Gucy1b1*^+/*LoxP*^ mice and Pf4-*Cre*^+^*Gucy1b1*^*LoxP*/*LoxP*^ mice was collected in heparinized tubes, gently diluted in PBS and centrifuged for 10 min at 100*g* and room temperature without active deceleration. PRP was subjected to a second centrifugation step at 700*g* to obtain platelets that were resuspended in Roswell Park Memorial Institute (RPMI) 1640 medium (catalog no. A1049101; Thermo Fisher Scientific) and activated by orbital shaking for 30 min at 1,000 rpm. Samples were centrifuged at 12,000*g* for 10 min and the supernatant was directly used in subsequent experiments. Thrombocyte count was analyzed simultaneously with an automated hematology analyzer (Sysmex Corporation).

For sGC stimulation, platelet suspension of WT mice was split in 2 vials containing either BAY-747 (150 ppm (150 mg l^−1^) final concentration) or vehicle (DMSO, 0.4%), mixed gently and incubated for 30 min before shaking.

Blood from healthy volunteers was collected in hirudin-coated tubes (Sarstedt) and centrifuged for 13 min at 170 g and room temperature without active deceleration. PRP was transferred into new tubes and activated by orbital shaking for 30 min at 1,000 rpm. Afterwards, samples were centrifuged at 12,000 *g* for 10 min and the supernatant was stored at −80 °C before conducting subsequent experiments. Thrombocyte count was determined from PRP as stated above.

### In vitro leukocyte adhesion

Bone marrow monocytes and neutrophils were isolated from C57BL/6J mice using magnetic-activated cell sorting cell separation columns (catalog no. 130-042-401; Miltenyi Biotec) after incubation with either anti-Ly6G-PE (clone 1A8; catalog no. 127608) or anti-CD115-biotin (clone AFS98; catalog no. 135508, both BioLegend and 1:200) antibodies followed by phycoerythrin (PE)- and streptavidin-coated microbeads (catalog nos. 130-048-801 and 130-048-101; Miltenyi Biotec), respectively.

Primary murine aortic ECs (catalog no. C57–6052, mAoEC; Cell Biologics) were cultured in complete EC medium (catalog no. PB-M1168; PeloBiotech) in a humidified incubator with 5% CO_2_ at 37 °C and grown to confluency in 96-well plates for experiments.

Adhesion assays were performed using the CytoSelect Leukocyte-Endothelium Adhesion Assay (catalog no. CBA-210; Cell Biolabs) according to the manufacturer’s instructions. Briefly, leukocytes were fluorescently labeled with LeukoTracker solution, resuspended in RPMI 1640 and added to mAoECs to a number of 2.5 × 10^5^ cells. Cells were incubated for 1 h at 37 °C in the presence of 50 μl of activated platelet supernatant. Plates were washed three times to remove nonadherent cells, lysed in 1× lysis buffer and fluorescence was measured on an Infinite M200 PRO plate reader (Exc = 485 nm and Em = 535 nm; TECAN). Experiments were conducted in triplicate.

For the preincubation experiments, either neutrophils/monocytes or ECs were exclusively incubated with activated platelet supernatant of WT mice for 30 min before performing the adhesion assay, omitting the additional administration of platelet supernatant in this step.

To inhibit the Tie2 receptor effects, mAoECs were preincubated with 0.5 μM BAY-826 (catalog no. 6579; R&D Systems) or vehicle (DMSO, 0.1%) for 5 h before performing the adhesion assay.

### Incubation of ECs with activated platelet supernatant

mAoECs were grown to confluence in 12-well dishes and stimulated with activated platelet supernatant from either Pf4-*Cre*^+^*Gucy1b1*^+/*LoxP*^ or Pf4-*Cre*^+^*Gucy1b1*^*LoxP*/*LoxP*^ mice in RPMI 1640 for 1 h at 37 °C. Cells were lysed by adding 500 μl TRIzol and stored at −80 °C.

### Intravital fluorescence microscopy and in vivo leukocyte adhesion

Pf4-*Cre*^+^*Gucy1b1*^+/*LoxP*^*Ldlr*^−/−^ and Pf4-*Cre*^+^*Gucy1b1*^*LoxP*/*LoxP*^*Ldlr*^−/−^ mice were anesthetized with a combination of midazolam, medetomidine and fentanyl and subjected to intravital microscopy of the right carotid artery bifurcation as described previously^49^. Leukocytes were labeled in vivo by intravenous injection of anti-Ly6G (PE-conjugated, clone 1A8, catalog no. 127608), anti-Ly6C (Alexa Fluor 488-conjugated, clone HK1.4, catalog no. 128022) and anti-CD11b (PE-conjugated, clone M1/70, catalog no. 101225; all BioLegend) antibodies (1 μg each per mouse). To examine leukocyte to endothelium interactions, movies of 30 s each were acquired using an Olympus BX51 microscope with a Hamamatsu 9100–02 electron-multiplying charge-coupled device camera (Hamamatsu) and a 10× saline-immersion objective. In subsequent analysis of the movies, the number of adherent neutrophils (Ly6G-PE^+^), monocytes (Ly6C-Alexa Fluor 488^+^), and myeloid cells (CD11b-PE^+^) was examined in a blinded manner, considering cells as adherent if their position did not change during imaging.

### Cytokine profiling and enzyme-linked immunosorbent assays

Cytokine profiling assays were performed using the Proteome Profiler Mouse XL Cytokine Array (catalo no. ARY028; R&D Systems) according to the manufacturer’s protocol. Briefly, after isolating and activating the platelets of Pf4-*Cre*^+^*Gucy1b1*^+/*LoxP*^ and Pf4-*Cre*^+^*Gucy1b1*^*LoxP*/*LoxP*^ mice by shaking in RPMI as described above, samples were added to the supplied antibody-spotted nitrocellulose membrane and incubated at 4 °C overnight. Captured proteins were detected using a mixture of biotinylated detection antibodies followed by streptavidin-HRP and visualized using chemiluminescent detection reagents. Signal intensities were detected by an ImageQuant LAS 4000 imaging system and analyzed using the appropriate image analysis software (ImageQuant LAS TL, version 8.1; GE Healthcare Life Sciences). The signal intensities of target proteins were normalized to the signal intensities for the reference spots in each sample. The procedure was repeated for a total of six samples per group.

ANGPT1 ELISAs were performed to determine murine (catalog no. EK1296; Boster Biological Technology) and human (catalog no. DANG10; R&D Systems) protein levels according to the manufacturer’s recommendations.

### Coexpression analyses in STARNET

Aligned multitissue RNA-seq samples from STARNET^15^ were pseudolog transformed and normalized using L2 penalized regression with penalty term 1.0, adjusting for the covariates: sequencing laboratory; read length; RNA extraction protocol (PolyA and Ribo-Zero); age; and sex. Additional adjustments included the first four surrogate variables detected by surrogate variable analysis^50^ and flow cell information after singular value decomposition retaining components with eigenvalues >4. Coexpression modules were inferred using weighted gene coexpression network analysis^51^ with *β* = 5.2 for tissue-specific and *β* = 2.7 for cross-tissue correlations, resulting in both tissue-specific and cross-tissue coexpression networks as described previously^52^. These data and analyses were accessed through the STARNET browser (http://starnet.mssm.edu) by querying coexpression modules containing ANGPT1. KEGG pathway and GO enrichment was carried out on coexpression module 11 for transcripts derived from whole blood (1,012 out of 1,016 transcripts) using Enrichr^53^.

### Flow cytometry

Mice were killed under isoflurane anesthesia and blood was collected in EDTA-coated microvettes (Sarstedt). For in vivo staining of circulating blood leukocytes, an antibody directed against CD45-BV605 (1:10 in 100 μl PBS, clone 30-F11, catalog no. 103140; BioLegend) was injected intravenously 5 min before killing the animals. After lysing RBCs in 1× RBC lysis buffer, samples were washed and resuspended in fluorescence-activated cell sorting (FACS) buffer (PBS containing 0.5% BSA). Aortas were perfused through the left ventricle with PBS and excised from root to common iliac artery bifurcation after removing perivascular fat and surrounding other tissue, minced using fine scissors and digested in 450 U ml^−1^ collagenase I (catalog no. C0130), 125 U ml^−1^ collagenase XI (catalog no. C7657), 60 U ml^−1^ DNase I (catalog no. D5319) and 60 U ml^−1^ hyaluronidase (catalog no. H3506; all Sigma-Aldrich) for 1 h at 37 °C under agitation. Cell suspensions were filtered through 40-μm nylon cell strainers (BD Biosciences), washed and resuspended in FACS buffer.

Both blood and aortic cells were first stained for hematopoietic lineage markers with PE-conjugated antimouse antibodies directed against B220 (1:600, clone RA3–6B2, catalog no. 103208), CD49b (1:1200, clone DX5, catalog no.108908), CD90.2 (1:3,000, clone 53–2.1, catalog no. 140308), NK1.1 (1:600, clone PK136, catalog no. 108708), Ter119 (1:600, clone TER-119, catalog no. 116208), and Ly6G (1:600, clone 1A8, catalog no. 127608) for 15 min at 4 °C and washed. This was followed by a second round of staining for Ly6C-BV421 (clone HK4.1, catalog no. 128031), CD115-BV510 (clone AFS98, catalog no. 750893; BD Biosciences), CD45.2-PerCP/Cy5.5 (1:300, clone 104, catalog no. 109827), F4/80-PE-Cy7 (clone BM8, catalog no. 123113), and CD11b-APC-Cy7 (clone M1/70, catalog no. 101225) in 1:600 dilution, if not stated otherwise. Antibodies were purchased from BioLegend if not stated otherwise and validated by the manufacturers for use in flow cytometry.

Cells were submitted to flow cytometry analysis on a BD LSRFortessa (BD Biosciences) and analyzed with the FlowJo software v.9.9.6 (FlowJo LLC). Cells were gated on viable (forward scatter area (FSC-A) versus side scatter area (SSC-A)) and single (FSC-A versus forward scatter width (FSC-W) and SSC-A versus side scatter width) cells. Neutrophils were identified as lineage^high^(CD45.2/CD11b)^high^CD115^low^. Monocytes were identified as lineage^low^(CD45.2/CD11b/CD115)^high^Ly6C^high/low^. Macrophages were identified as lineage^low^(CD45.2/CD11b/F4/80)^high^Ly6C^low/int^.

### PamGene serine/threonine kinase array

Washed platelets from WT mice were incubated with vehicle or BAY-747 (150 ppm) and activated by shaking as described above. Cells were centrifuged at 1,200*g* for 5 min and resuspended in ice-cold M-PER lysis buffer (catalog no. 78503) supplemented with protease inhibitor (1:100) and phosphatase inhibitor (1:100, catalog no. 78428; all Thermo Fisher Scientific) to a concentration of 200 × 10^6^ platelets per 100 μl. Platelets were lysed for 15 min on ice. Lysates were centrifuged at 20,000*g* for 15 min and stored at −80 °C. Serine/threonine kinase profiles were determined using the PamChip serine/threonine kinase assay (PamGene International) as described previously^54^.

### Inhibition of platelet signaling pathways

Washed platelets were isolated from WT mice as described above and incubated with different inhibitors of downstream cGMP signaling (Supplementary Table 1) compared to vehicle for 30 min at room temperature. Subsequently, platelets were activated by shaking for 30 min, centrifuged, and supernatant was collected for determination of ANGPT1 release as described above.

### Determination of BAY-747 serum/plasma concentration

BAY-747 exposure was quantified in plasma using a liquid chromatography (LC) system for mass separation (Kinetex 5 μm C18 100 A LC Column 150 × 4.6 mm) coupled to a Triple Quad 4500 LC–mass spectrometry analyzer (positive mode; AB Sciex). A generic internal standard was added to the samples. A five-point calibration curve and quality control samples were used for relative quantification. Plasma was obtained from six mice per group. Data are the mean + s.e.m.

### Adoptive transfer of leukocytes

Bone marrow monocytes and neutrophils were isolated simultaneously from *Ubc-GFP* mice using anti-Ly6G-PE and anti-CD115-biotin antibodies followed by PE- and streptavidin-coated microbeads as stated above and resuspended in PBS. We intravenously injected equal amounts of isolated cells into *Ldlr*^−/−^ mice fed a 0 or 150 ppm BAY-747 containing Western diet for 6 weeks and collected blood and aortae 24 h later as stated above. The number of CD45.2^high^/CD11b^high^/GFP^high^ cells within the aorta normalized to the exact number of injected cells was quantified by flow cytometry.

### Statistical analysis

Normality distribution of the data was assessed using the Kolmogorov–Smirnov test or the Shapiro–Wilk test for sample sizes *n* < 5. Test results and subsequently used statistical tests are displayed in Supplementary Table 2. Data were analyzed using a two-tailed Student’s unpaired or paired *t*-test (for normally distributed data) or Mann–Whitney *U*-test (for non-normally distributed data), as appropriate and indicated in the respective figure legend and Supplementary Table 2. When comparing more than two groups, a repeated measures one-way analysis of variance (ANOVA) test followed by a Tukey test for multiple comparisons or mixed-effect analyses/repeated measures one-way ANOVA were performed, as appropriate, when data were normally distributed. To determine the statistical outliers, the two-sided ROUT test was used. If outliers were removed from the analysis, this is indicated in the respective figure legend. Sample sizes/numbers of replicates are indicated in the figure legends and visualized in the figures (each symbol represents one animal or biological replicate) and data are displayed as the mean + s.e.m. *P* values <0.05, when investigating more than two groups after adjustment for multiple testing, were regarded as statistically significant. Statistical analyses were performed with Prism v.9 for macOS (GraphPad Software).

## Extended Data

## Supplementary Material

Supplementary Tables

Source Data

## Data Availability

The data supporting the findings of this study are available within the paper and its extended data/supplementary material. The STARNET data are available at the database of Genotypes and Phenotypes (dbGaP) site (dbGaP study accession no. phs001203.v1.p1).
